# Supplementary material for: Neonatal AAV gene therapy rescues hearing in a mouse model of SYNE4 deafness
Source: EMBO Mol Med. 2020 Dec 22;13(2):e13259. doi: 10.15252/emmm.202013259 (PMC7863404; doi:10.15252/emmm.202013259)
Supplement: Supplementary file 2 — Expanded View Figures PDF [file EMMM-13-e13259-s002.pdf]

Expanded View Figures

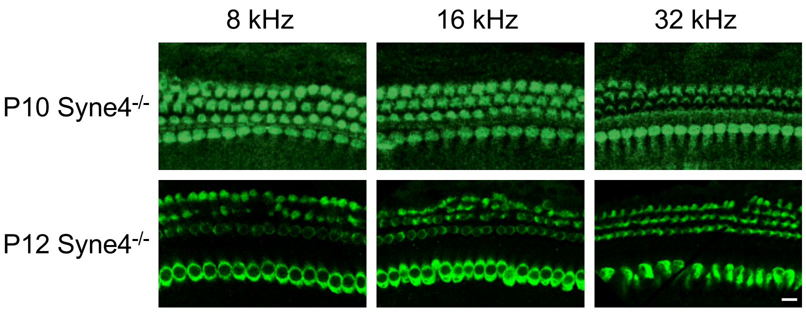

Figure EV1. *Syne4*$^{-/-}$ hair cells at P10 and P12.
Whole-mount immunofluorescence of *Syne4*$^{-/-}$ organ of Corti from the 8, 16, and 32 kHz regions at P10 and P12, labeled with myosin VIIa. Scale bar = 10 µm.

Source data are available online for this figure.

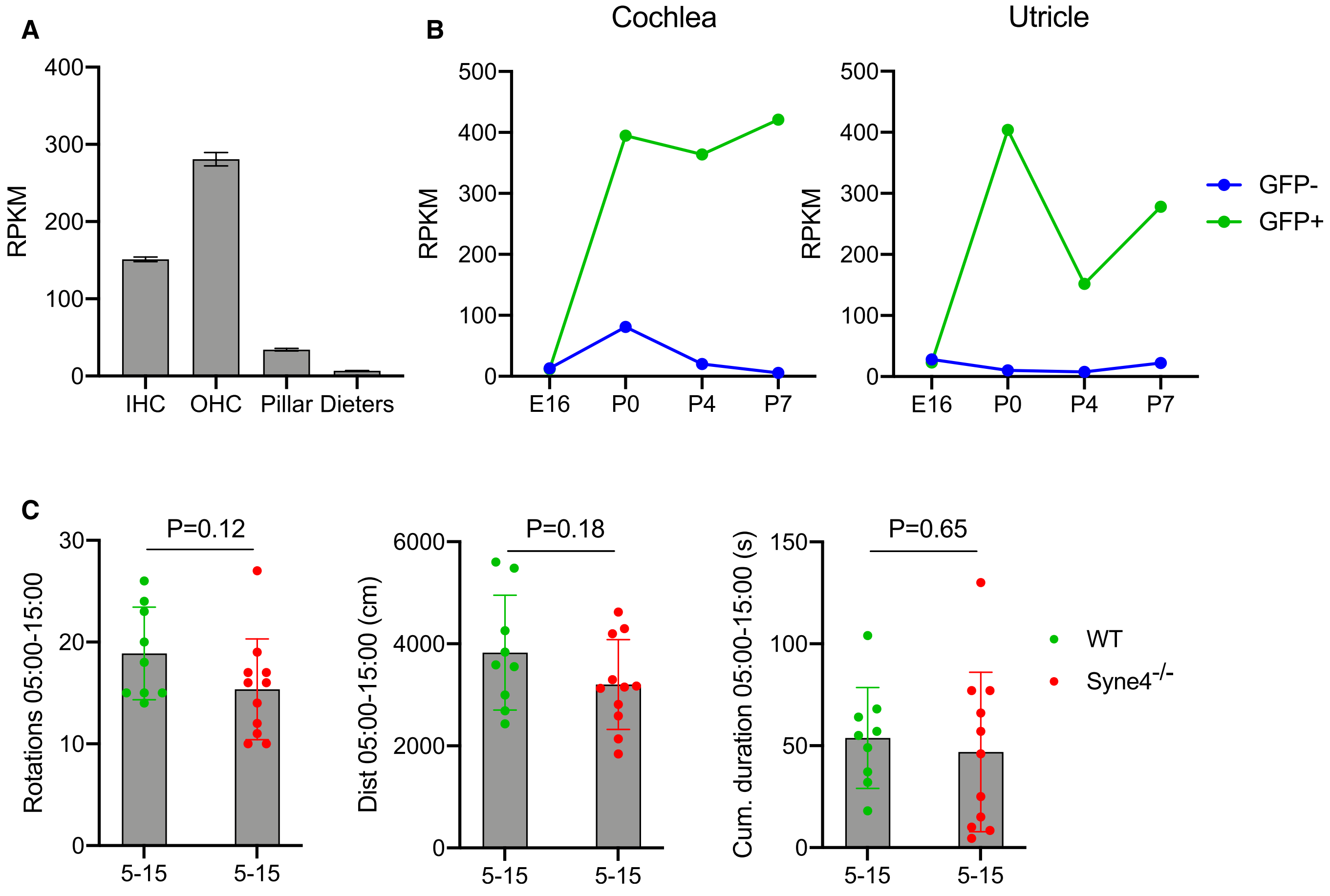

Figure EV2. Expression of *Syne4* in the inner ear and vestibular phenotype.

A *Syne4* expression in RNA-seq results from pooled P28-35 cells from CBA/J mice, n = 3 (Liu *et al*, 2018).

B *Syne4* expression in RNA-seq results from 16 samples of E16, P0, P4, and P7 Pou4f3-eGFP mice (Scheffer *et al*, 2015). Plots show average RPKM.

C Open-field results of WT and *Syne4*$^{-/-}$ mice at minutes 5–15 of the test, n = 9 for WT and n = 11 for *Syne4*$^{-/-}$. Statistical test was unpaired Student's t-test.

Data information: Plots show mean ± SD.
Source data are available online for this figure.

 © 2021 The Authors

Figure EV3. Safety profile characterization of AAV.Syne4.

A ABR thresholds at 4w of WT mice injected at P1 with AAV.Syne4 and un-injected controls, $n = 7$ for WT and $n = 10$ for WT + AAV.Syne4.
B ABR thresholds at 8w of WT mice injected at P1 with AAV.Syne4 and un-injected controls, $n = 6$ for WT and $n = 6$ for WT + AAV.Syne4.
C ABR thresholds at 12w of WT mice injected at P1 with AAV.Syne4 and un-injected controls, $n = 6$ for WT and $n = 5$ for WT + AAV.Syne4.
D DPOAE thresholds at 4w of WT mice injected at P1 with AAV.Syne4 and un-injected controls, $n = 6$ for WT and $n = 6$ for WT + AAV.Syne4.
E Open-field results of WT and WT + AAV.Syne4 mice at 12w at minutes 5-15 of the test, $n = 9$ for WT and $n = 7$ for WT + AAV.Syne4.
F Weight gain over time in the different groups tested, $n = 5$ for WT, $n = 5$ for *Syne4*$^{-/-}$, $n = 11$ for *Syne4*$^{-/-}$ + AAV.Syne4, $n = 6$ for WT + AAV.Syne4, and $n = 7$ for *Syne4*$^{-/-}$ + AAV.GFP.

Data information: Statistical tests were 2-way ANOVA for ABR and DPOAE with Holm–Sidak correction for multiple comparisons, Student's *t*-test for vestibular tests, and mixed-effects model for weight gain. Plots show mean ± SD. ns = not significant.
Source data are available online for this figure.

© 2021 The Authors

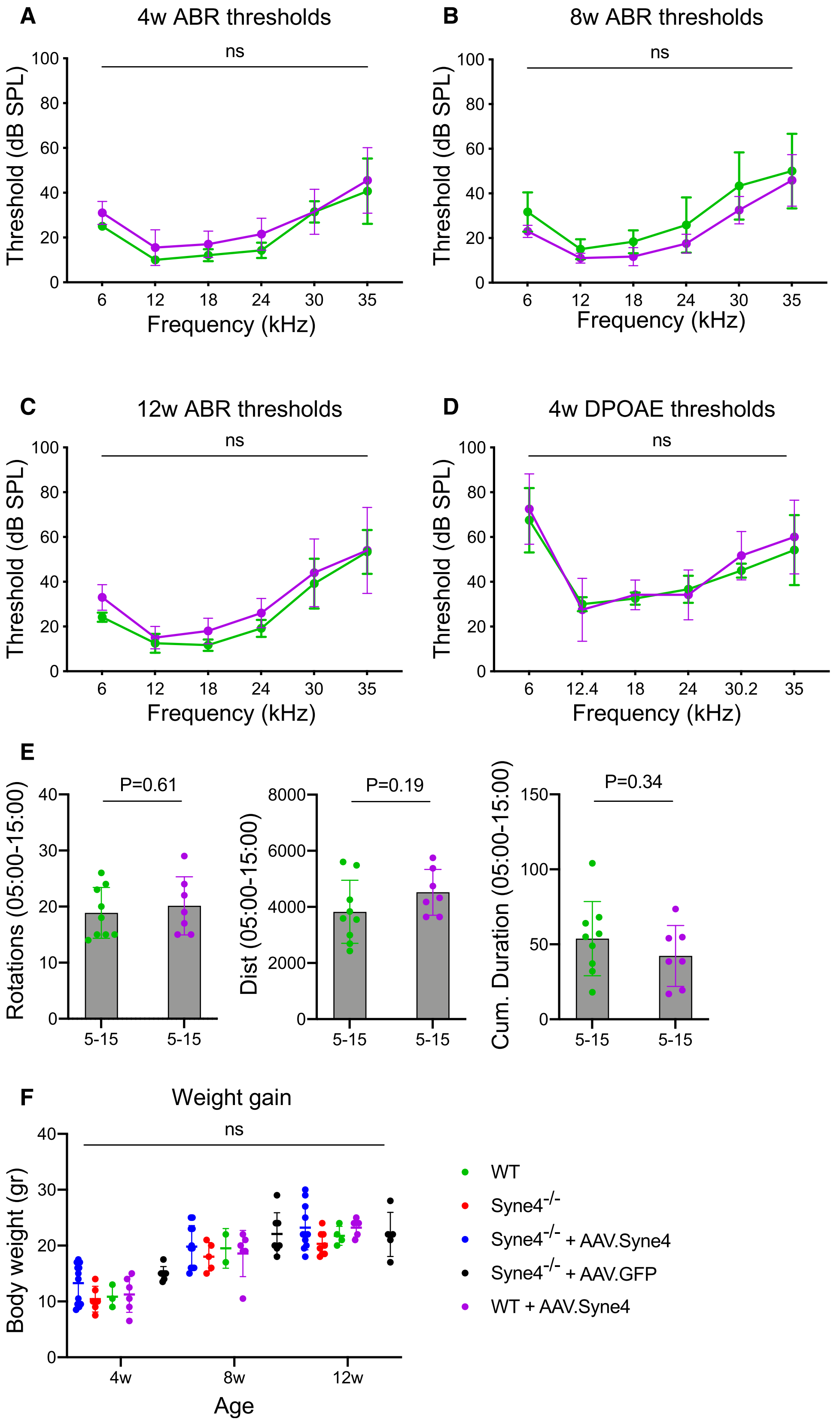

Figure EV3.

 © 2021 The Authors

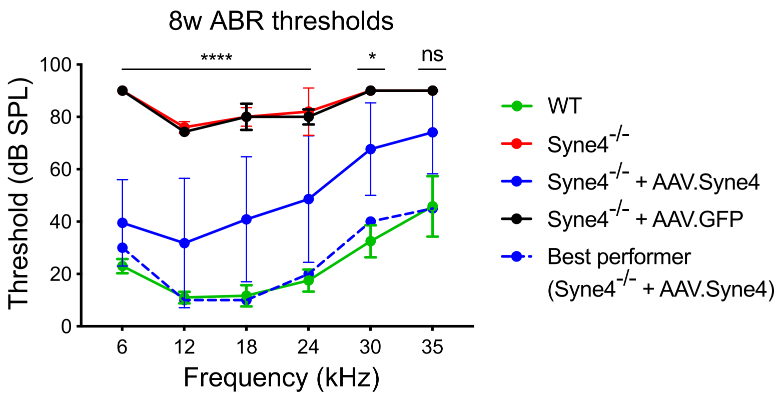

Figure EV4. ABR thresholds of injected *Syne4*$^{-/-}$ mice at 8w.

ABR thresholds at 8w of WT, *Syne4*$^{-/-}$, *Syne4*$^{-/-}$ mice injected with AAV.Syne4, and *Syne4*$^{-/-}$ mice injected with AAV.GFP, $n = 6$ for WT, $n = 5$ for *Syne4*$^{-/-}$, $n = 11$ for *Syne4*$^{-/-}$ + AAV.Syne4, and $n = 7$ for *Syne4*$^{-/-}$ + AAV.GFP. Statistical tests were 2-way ANOVA with Holm–Sidak correction for multiple comparisons. Plots show mean ± SD.
****$P < 0.0001$, *$P < 0.05$, ns = not significant.

Source data are available online for this figure.
