# Supplementary material for: Neonatal AAV gene therapy rescues hearing in a mouse model of SYNE4 deafness
Source: EMBO Mol Med. 2020 Dec 22;13(2):e13259. doi: 10.15252/emmm.202013259 (PMC7863404; doi:10.15252/emmm.202013259)
Supplement: Supplementary file 3 — Movie EV1 [file EMMM-13-e13259-s003.zip › Movie EV 1.docx]

**Movie EV 1. Representative examples of cued-fear conditioning results on day 2 of the procedure**

Cued-fear conditioning results from WT, *Syne4^-/-^* mice and *Syne4^-/-^* mice injected with AAV.Syne4. Red bars indicate detected freezing behavior, red line indicates detected activity level.
