# Supplementary figures and images for: Neonatal AAV gene therapy rescues hearing in a mouse model of SYNE4 deafness
Source: EMBO Mol Med. 2020 Dec 22;13(2):e13259. doi: 10.15252/emmm.202013259 (PMC7863404; doi:10.15252/emmm.202013259)

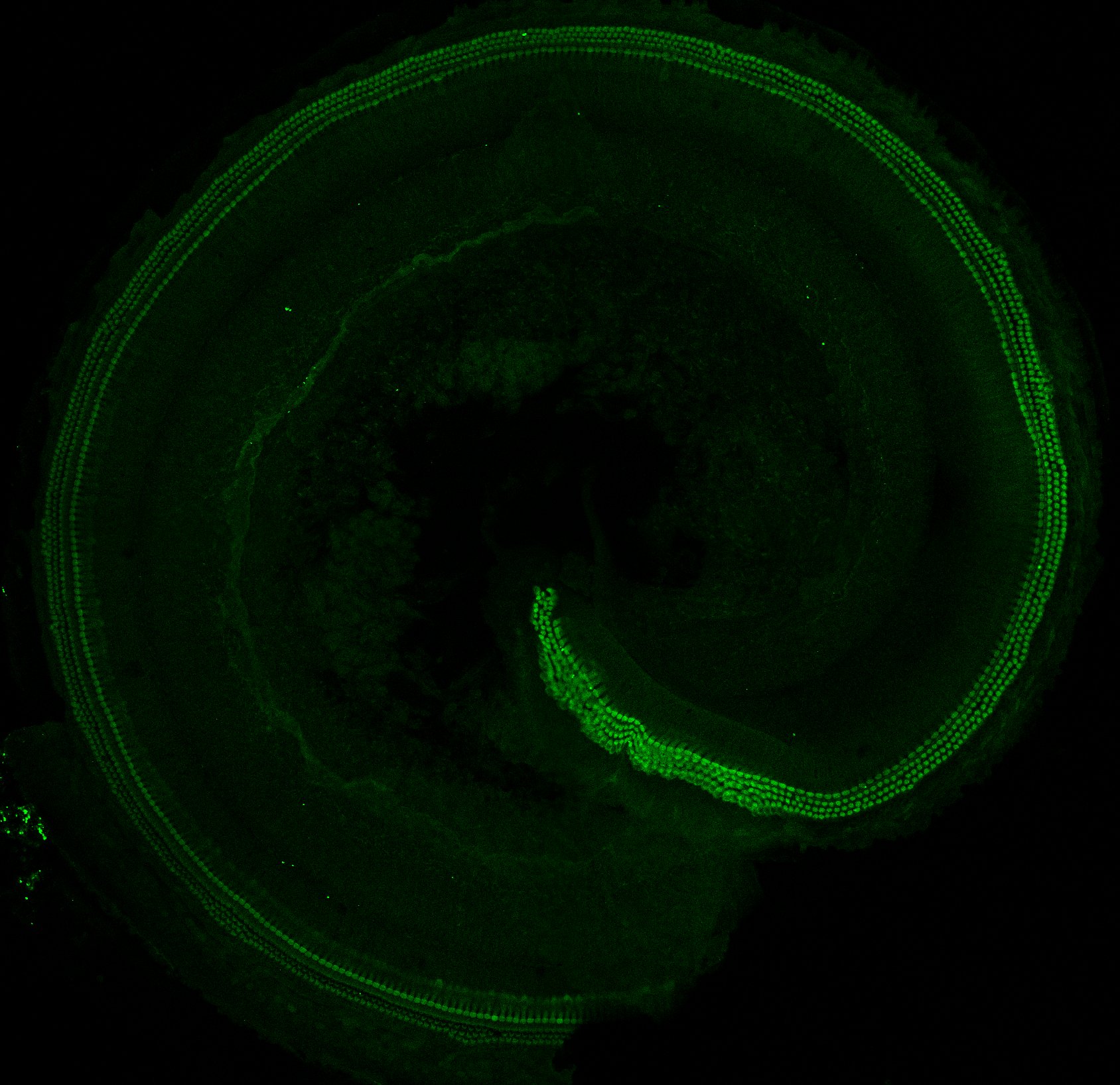

Supplement: Supplementary file 4 — Source Data for Expanded View [file EMMM-13-e13259-s010.zip › Fig EV1/P10 mut tile scan.jpg]

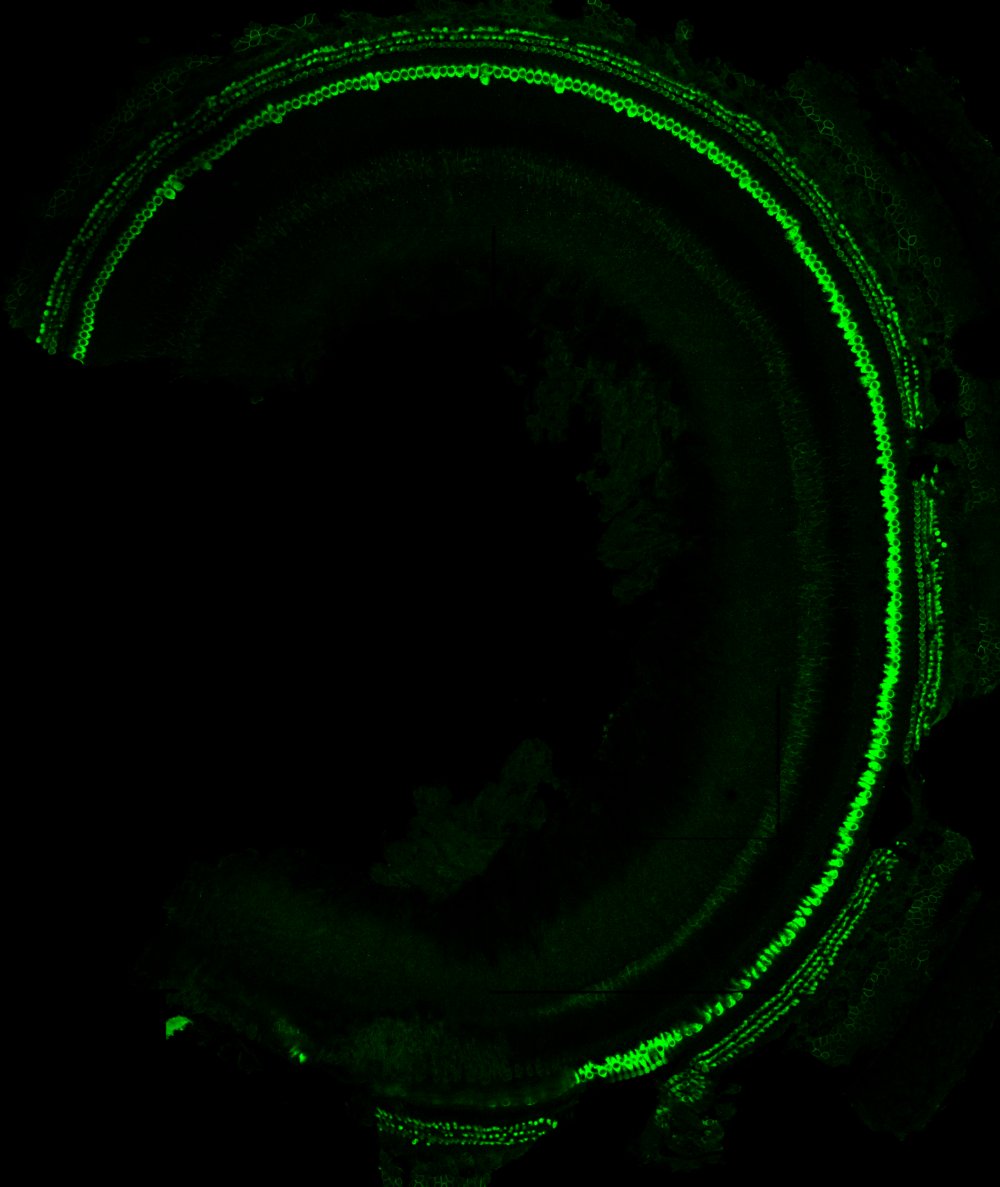

Supplement: Supplementary file 4 — Source Data for Expanded View [file EMMM-13-e13259-s010.zip › Fig EV1/P12 mut tile scan.jpg]

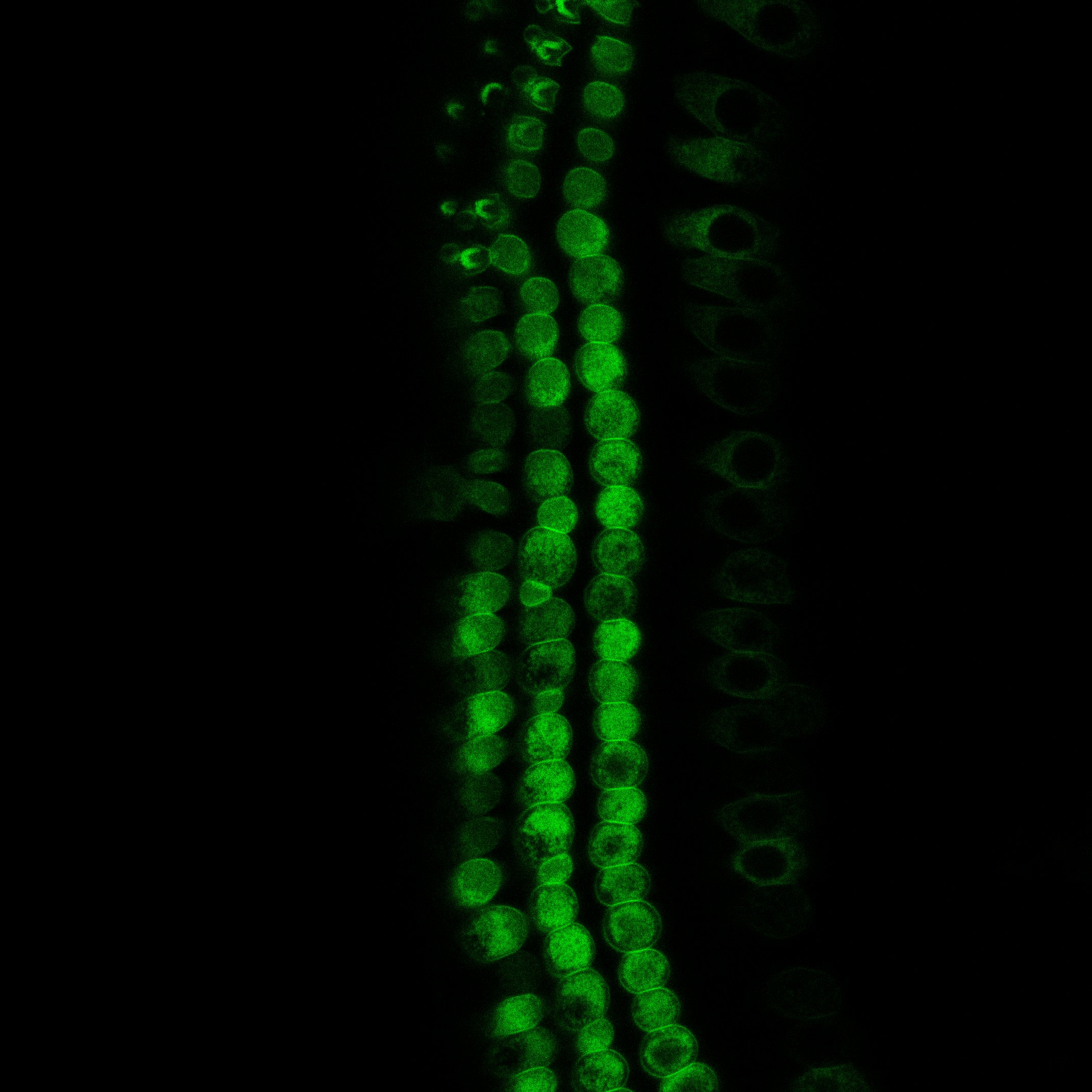

Supplement: Supplementary file 6 — Source Data for Figure 2 [file EMMM-13-e13259-s004.zip › Figure 2/P8+1DIV mut FM1-43.jpg]

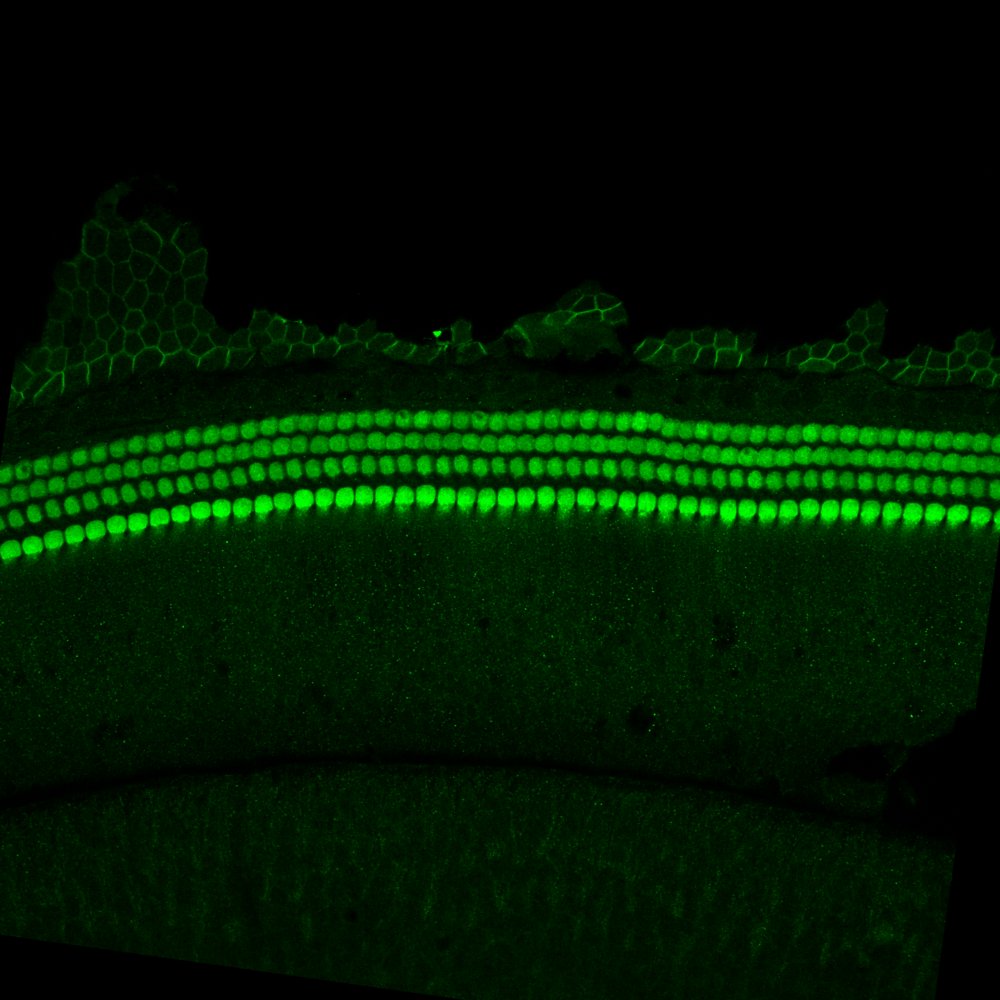

Supplement: Supplementary file 6 — Source Data for Figure 2 [file EMMM-13-e13259-s004.zip › Figure 2/P8 WT 16khz.jpg]

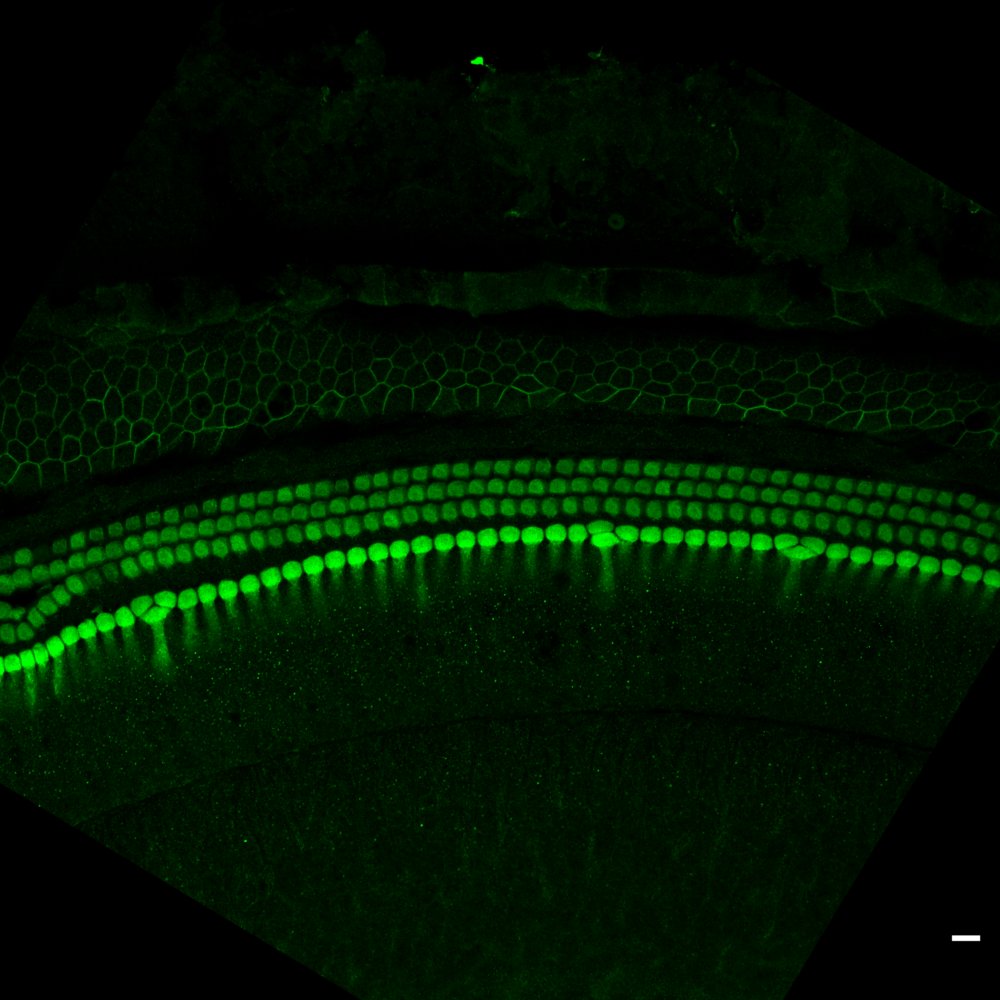

Supplement: Supplementary file 6 — Source Data for Figure 2 [file EMMM-13-e13259-s004.zip › Figure 2/P8 WT 32khz.jpg]

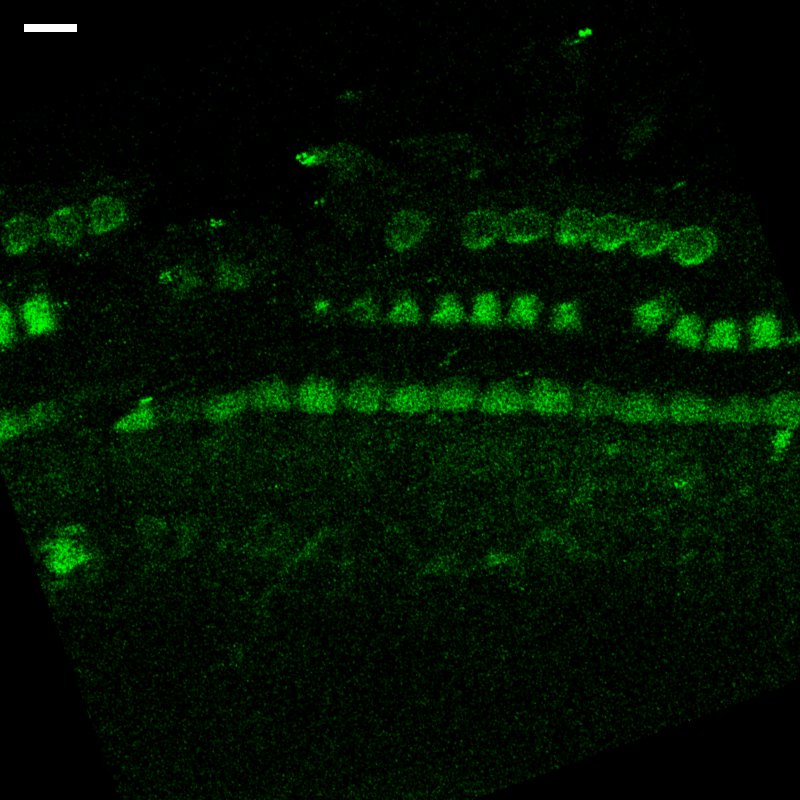

Supplement: Supplementary file 6 — Source Data for Figure 2 [file EMMM-13-e13259-s004.zip › Figure 2/P14 mut 16khz.jpg]

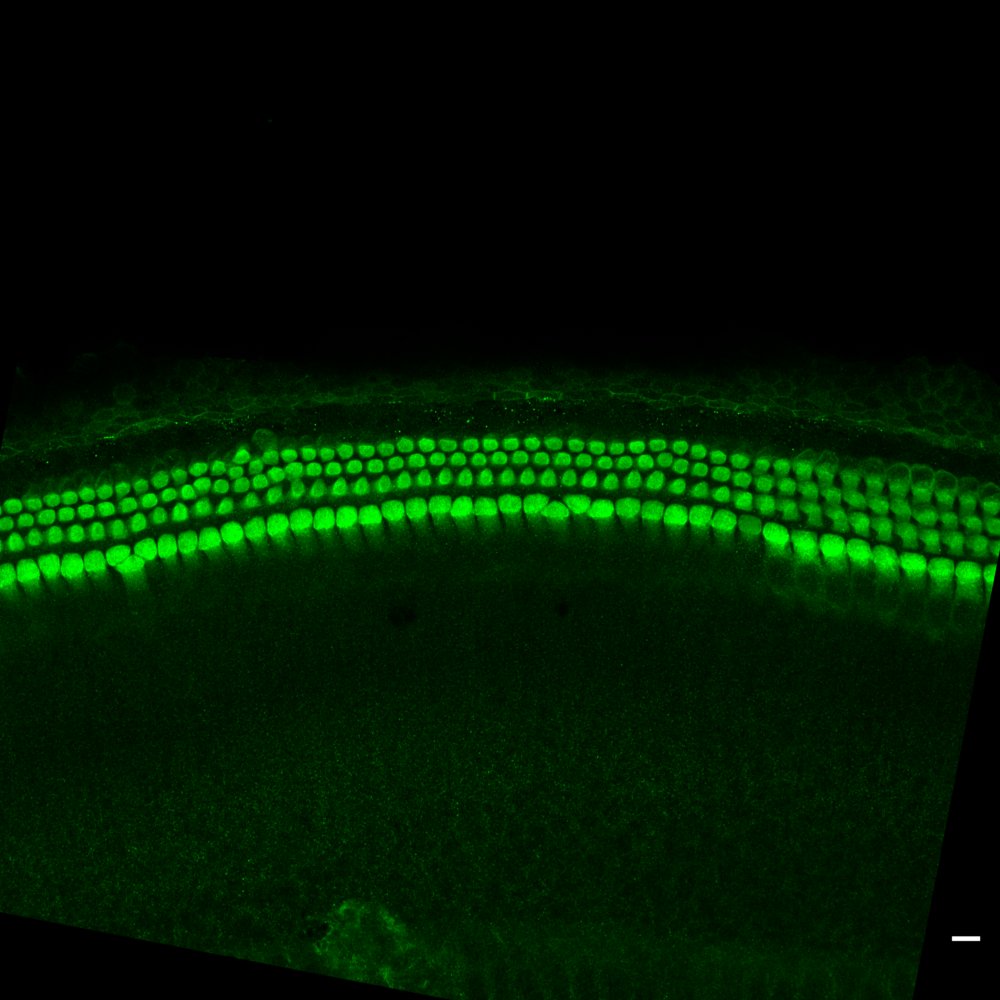

Supplement: Supplementary file 6 — Source Data for Figure 2 [file EMMM-13-e13259-s004.zip › Figure 2/P8 mut 16khz.jpg]

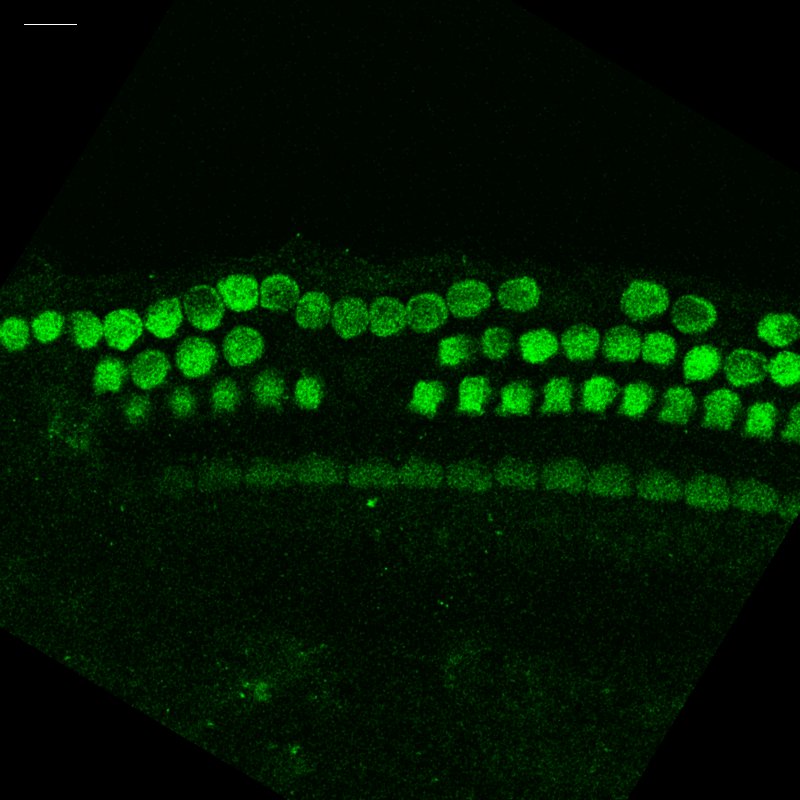

Supplement: Supplementary file 6 — Source Data for Figure 2 [file EMMM-13-e13259-s004.zip › Figure 2/P14 mut 8khz.jpg]

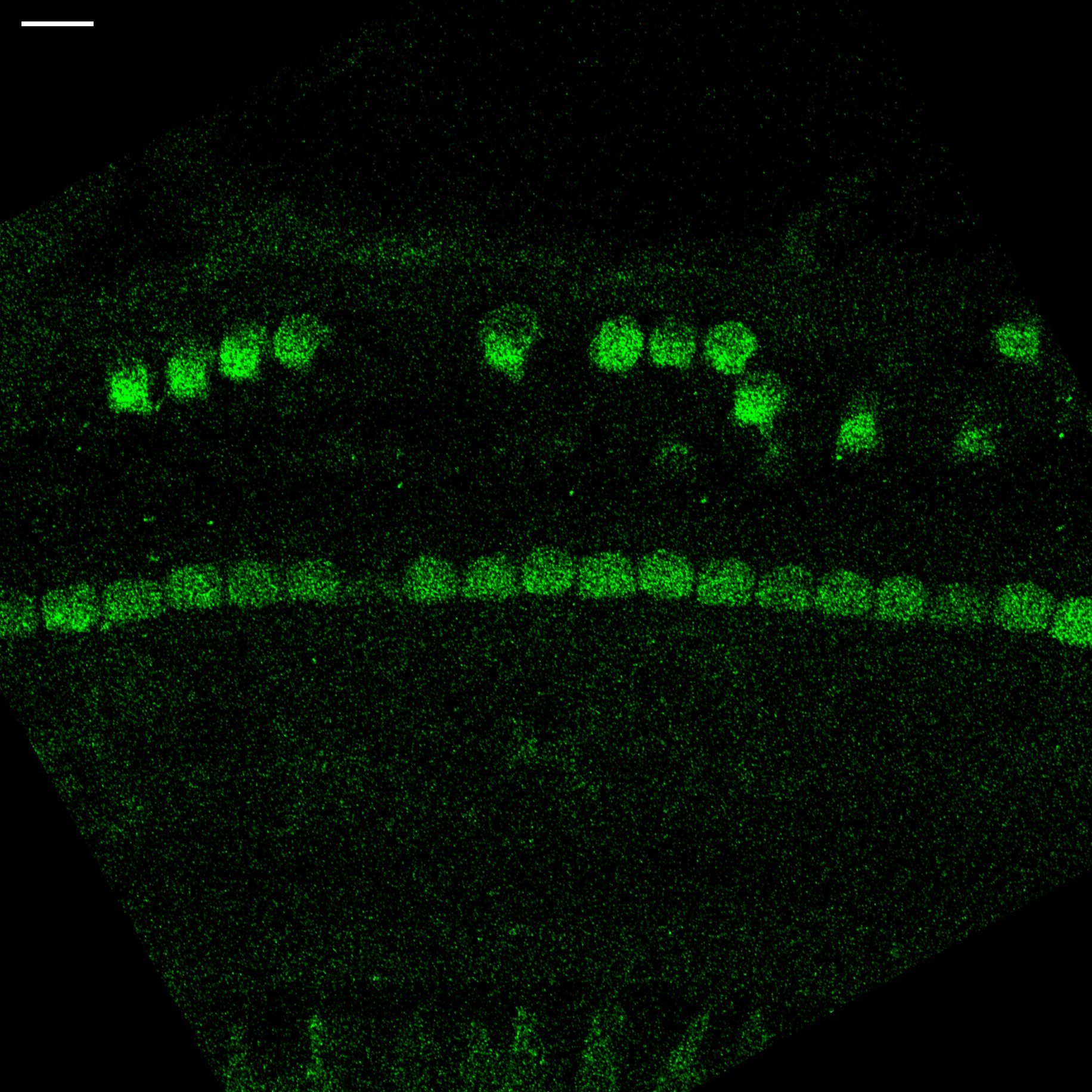

Supplement: Supplementary file 6 — Source Data for Figure 2 [file EMMM-13-e13259-s004.zip › Figure 2/P14 mut 32khz.jpg]

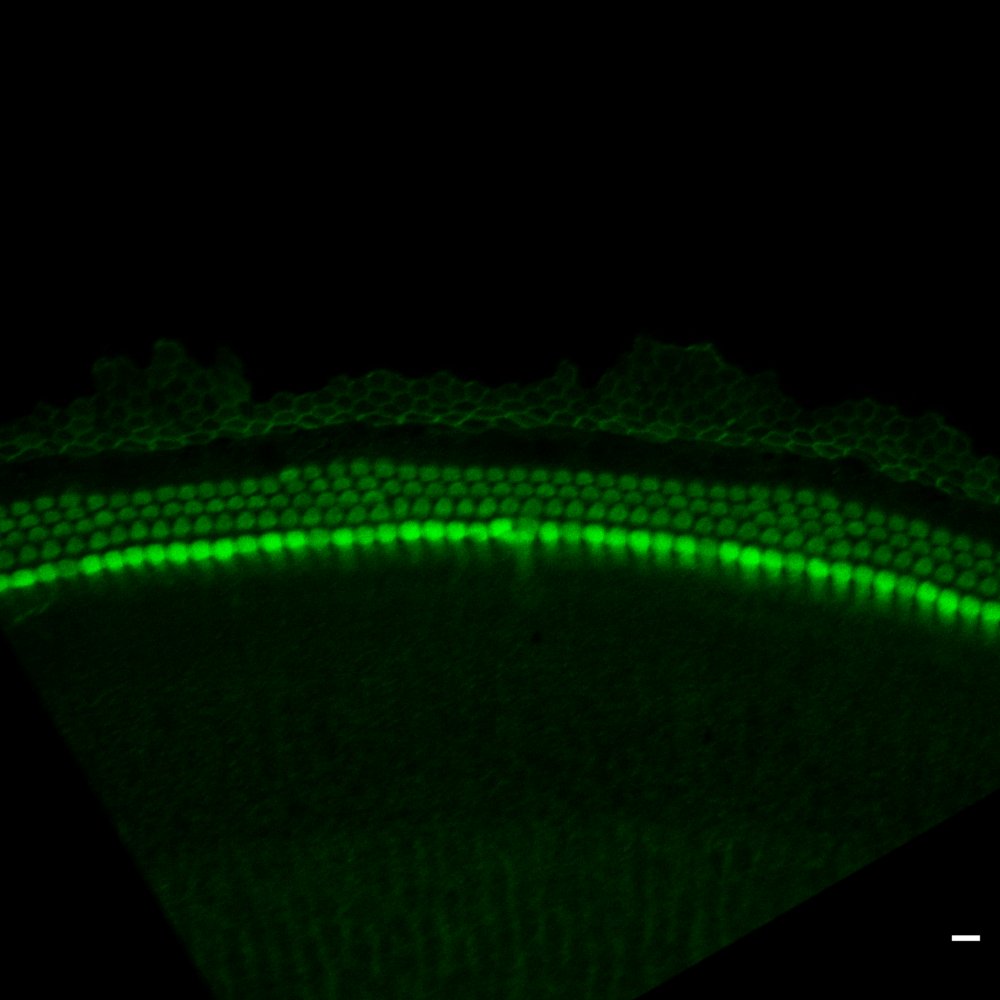

Supplement: Supplementary file 6 — Source Data for Figure 2 [file EMMM-13-e13259-s004.zip › Figure 2/P8 mut 32khz.jpg]

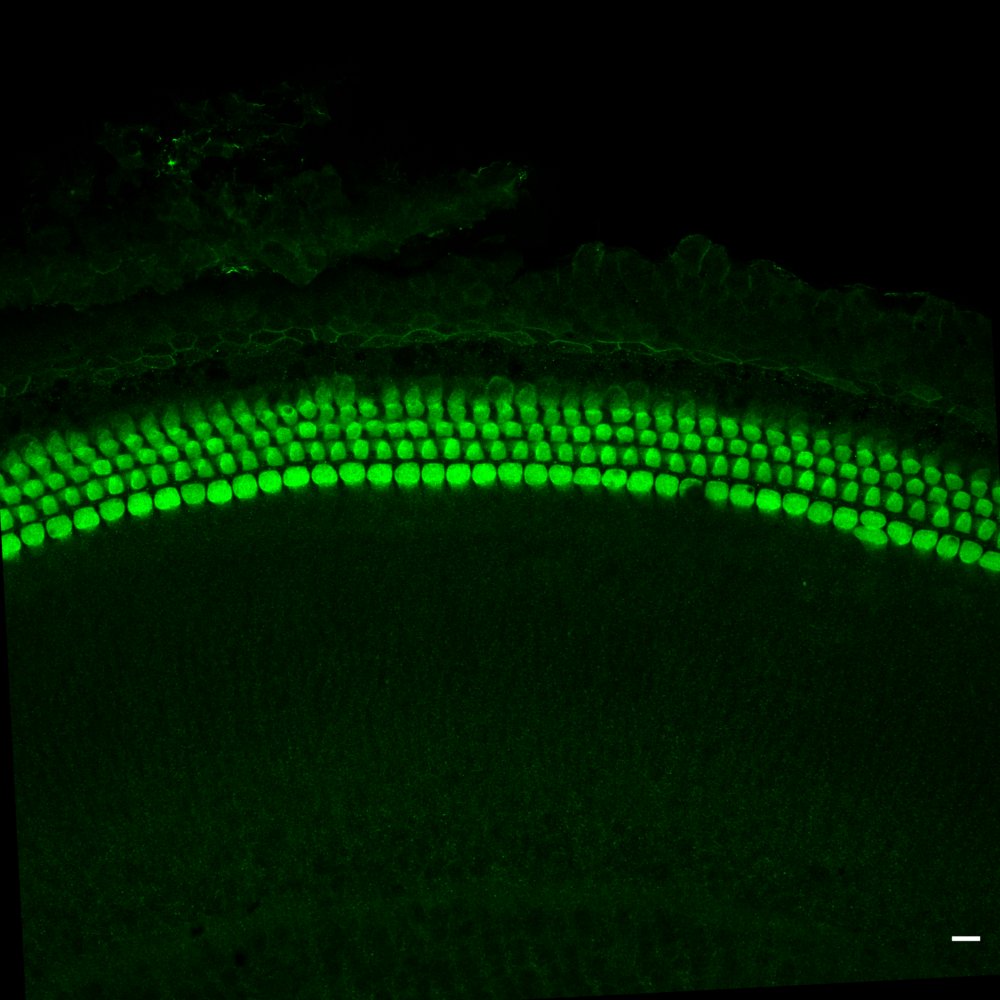

Supplement: Supplementary file 6 — Source Data for Figure 2 [file EMMM-13-e13259-s004.zip › Figure 2/P8 mut 8khz.jpg]

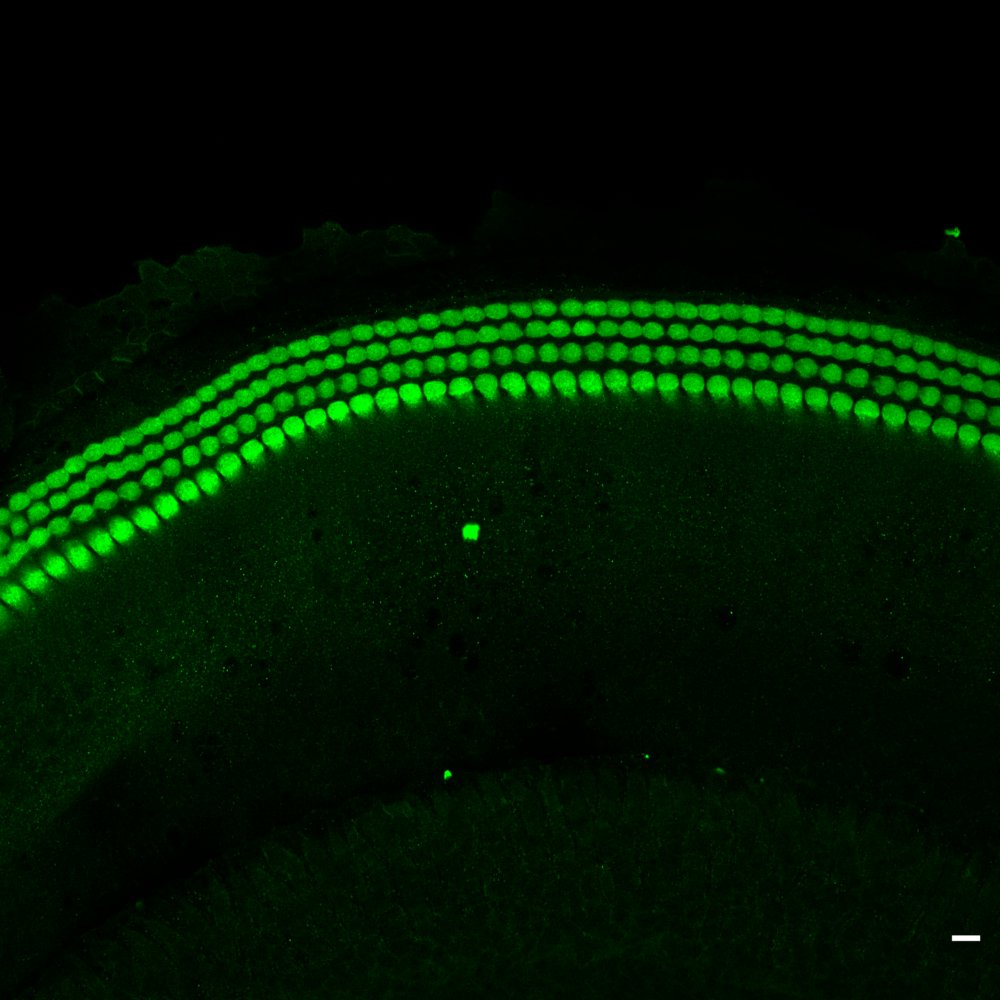

Supplement: Supplementary file 6 — Source Data for Figure 2 [file EMMM-13-e13259-s004.zip › Figure 2/P8 WT 8khz.jpg]

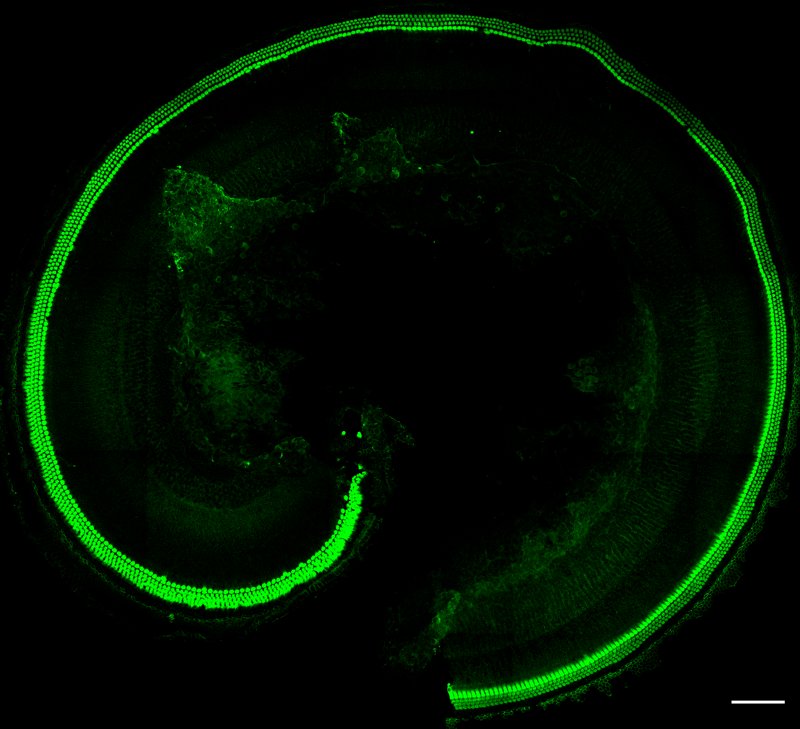

Supplement: Supplementary file 6 — Source Data for Figure 2 [file EMMM-13-e13259-s004.zip › Figure 2/P8 mut tile scan.jpg]

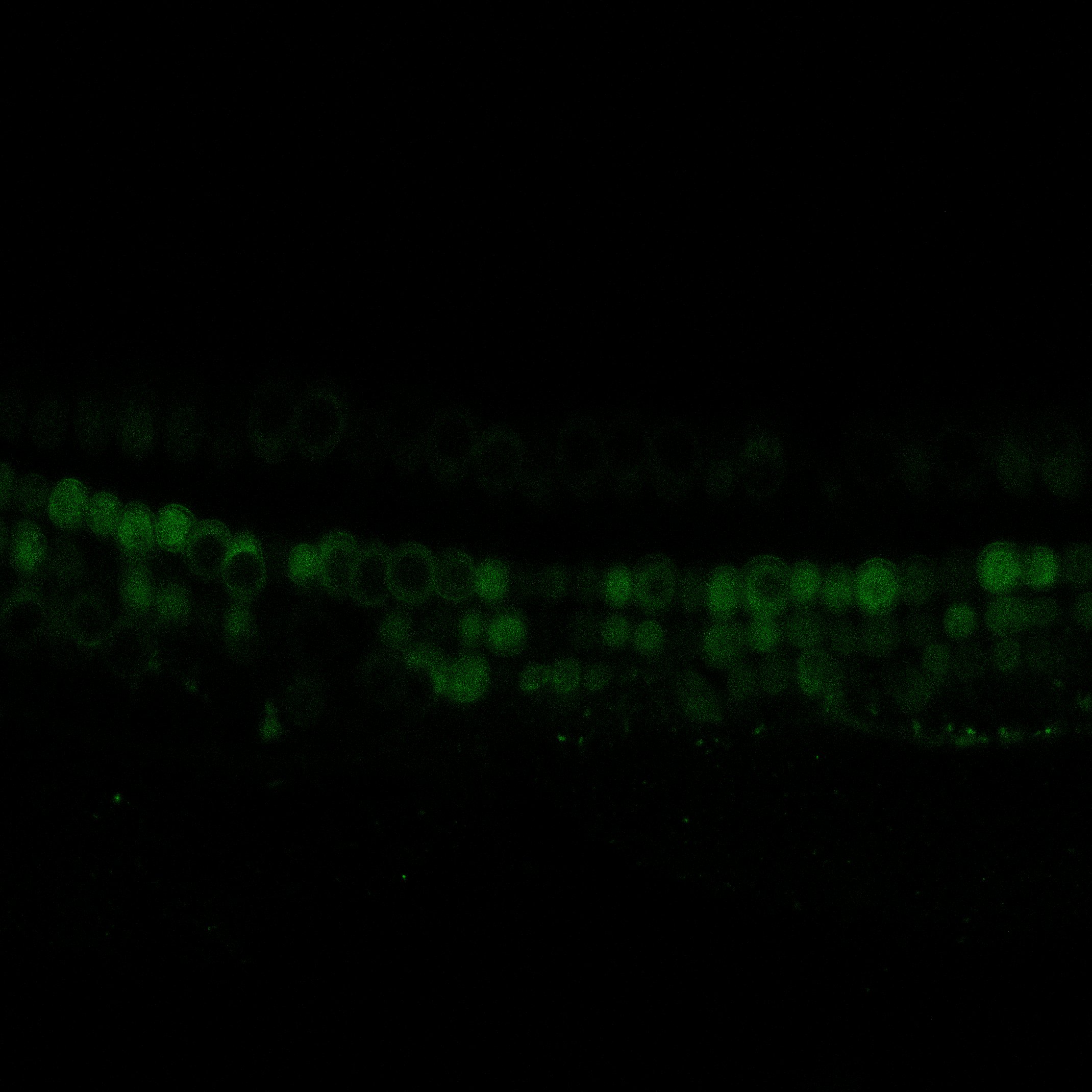

Supplement: Supplementary file 6 — Source Data for Figure 2 [file EMMM-13-e13259-s004.zip › Figure 2/P8+1DIV WT FM1-43.jpg]

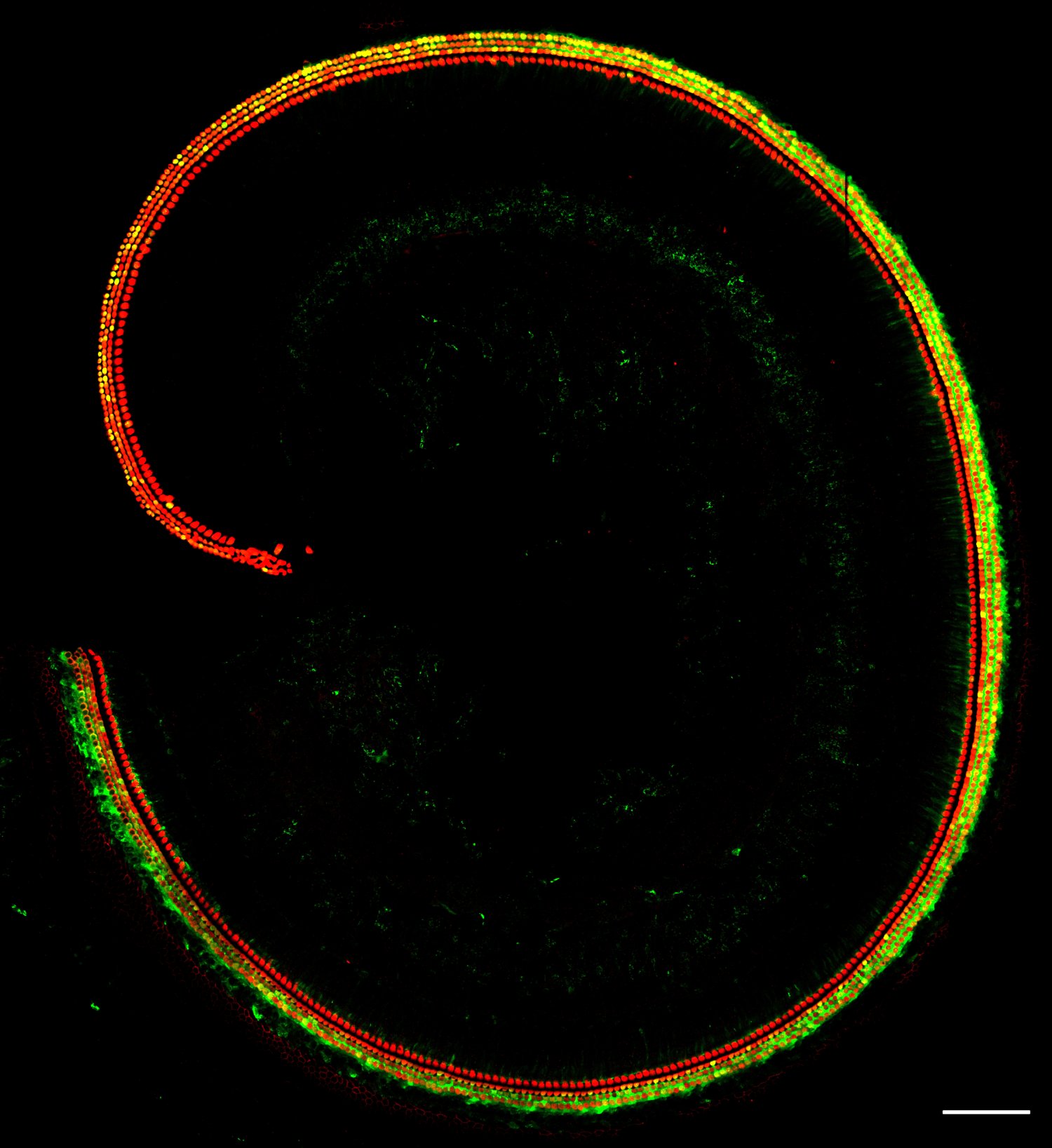

Supplement: Supplementary file 7 — Source Data for Figure 3 [file EMMM-13-e13259-s005.zip › Figure 3/P9 inj AAV-GFP tile can.jpg]

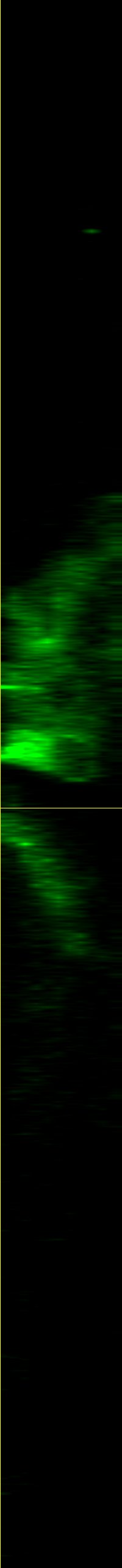

Supplement: Supplementary file 7 — Source Data for Figure 3 [file EMMM-13-e13259-s005.zip › Figure 3/YZ 927-1 8khz.jpg]

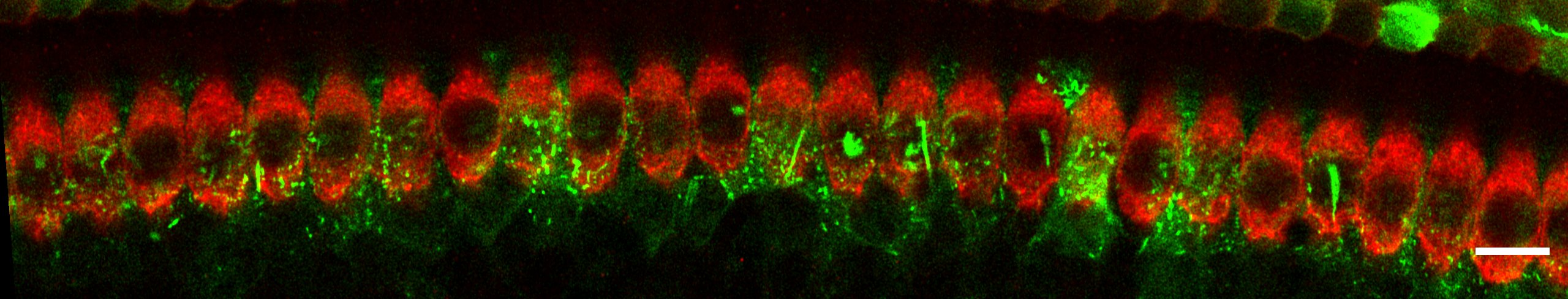

Supplement: Supplementary file 7 — Source Data for Figure 3 [file EMMM-13-e13259-s005.zip › Figure 3/GFP #3 b.27.05 ext 03.06 32khz-2 IHC.czi (RGB).jpg]

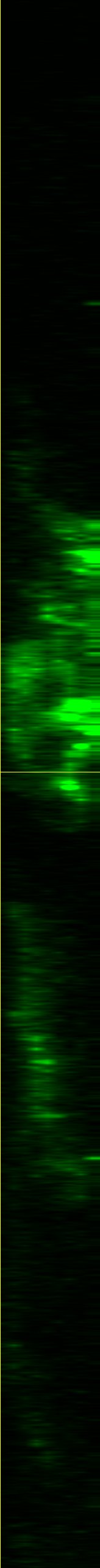

Supplement: Supplementary file 7 — Source Data for Figure 3 [file EMMM-13-e13259-s005.zip › Figure 3/YZ 1672-1 32khz.jpg]

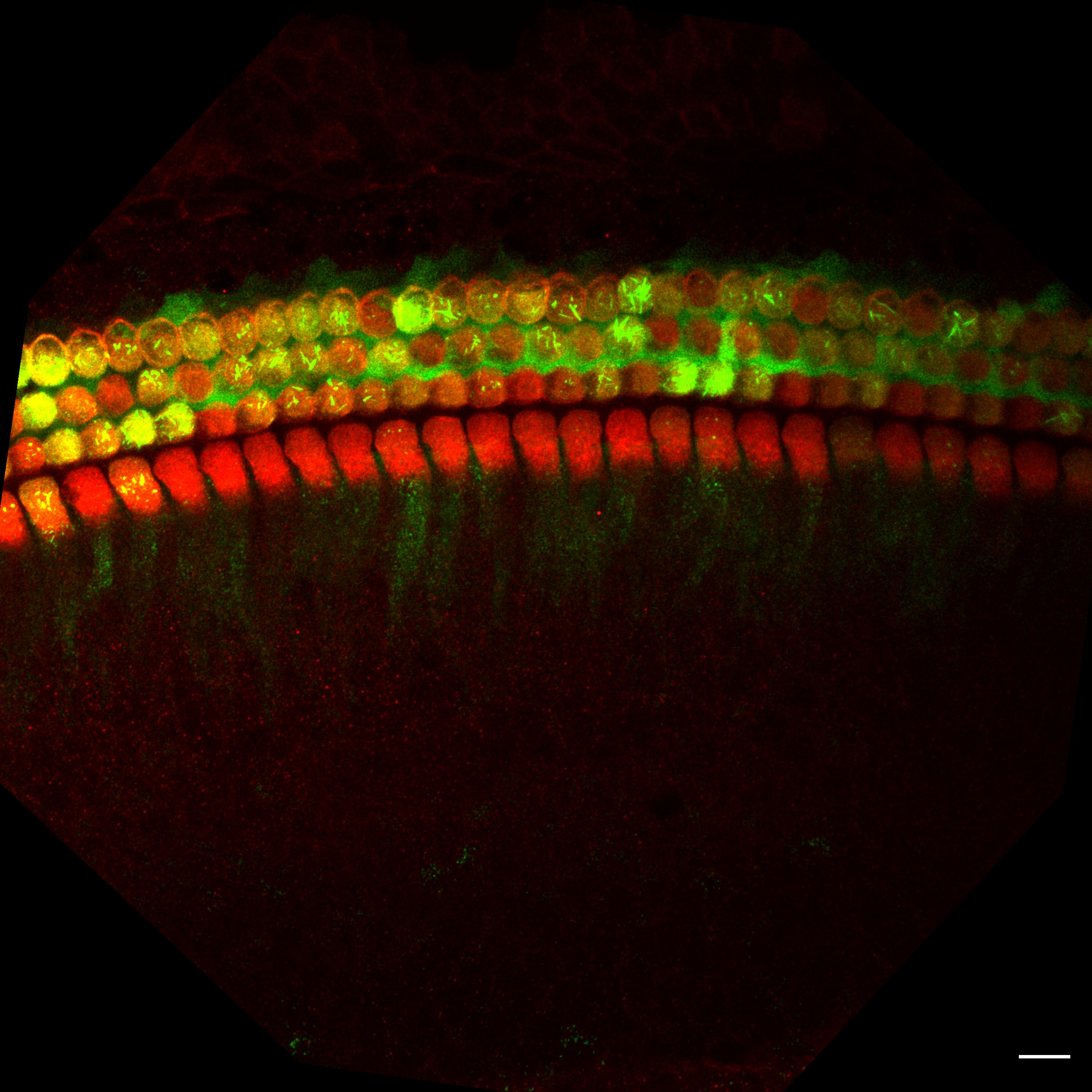

Supplement: Supplementary file 7 — Source Data for Figure 3 [file EMMM-13-e13259-s005.zip › Figure 3/P9 inj AAV-GFP 8khz.jpg]

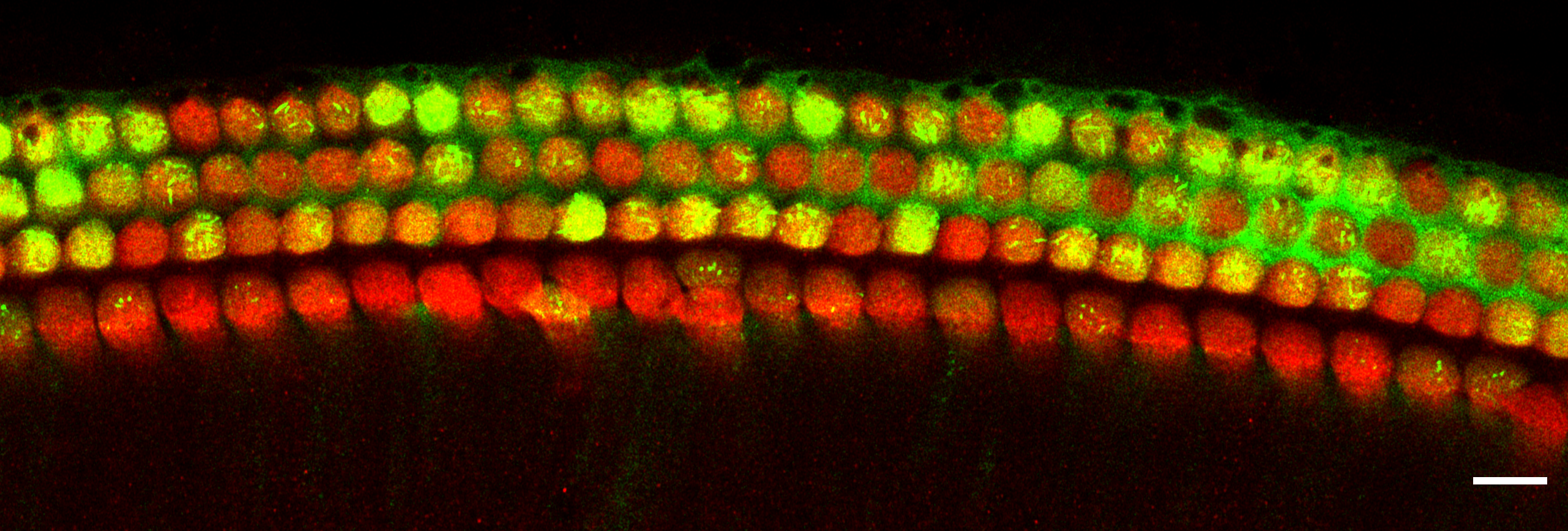

Supplement: Supplementary file 7 — Source Data for Figure 3 [file EMMM-13-e13259-s005.zip › Figure 3/GFP #1 b.27.05 ext 03.06 8khz-2 OHC.png]

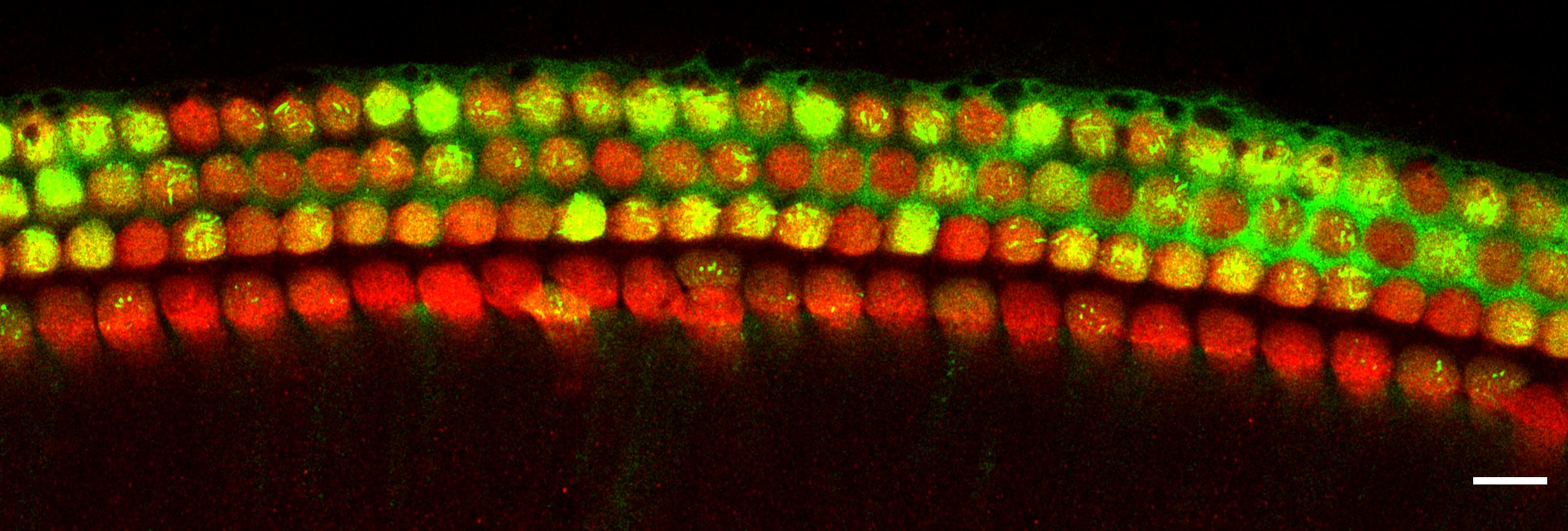

Supplement: Supplementary file 7 — Source Data for Figure 3 [file EMMM-13-e13259-s005.zip › Figure 3/GFP #1 b.27.05 ext 03.06 8khz-2 OHC.jpg]

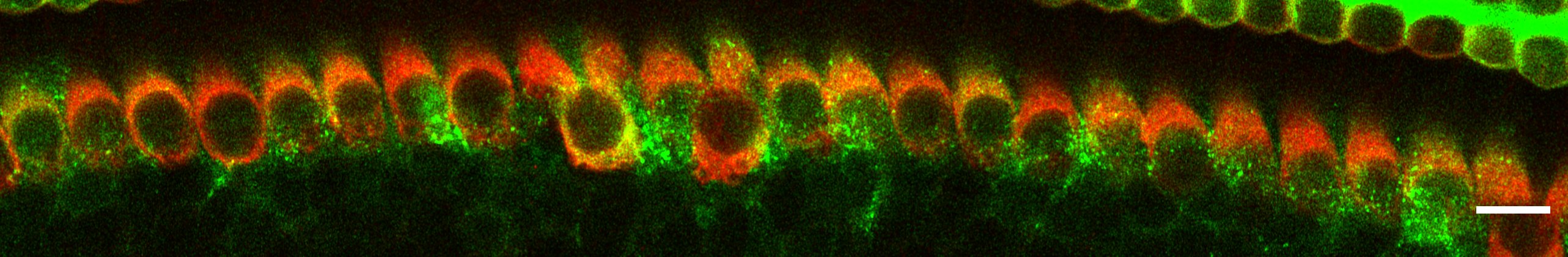

Supplement: Supplementary file 7 — Source Data for Figure 3 [file EMMM-13-e13259-s005.zip › Figure 3/GFP #1 b.27.05 ext 03.06 8khz-2 IHC.jpg]

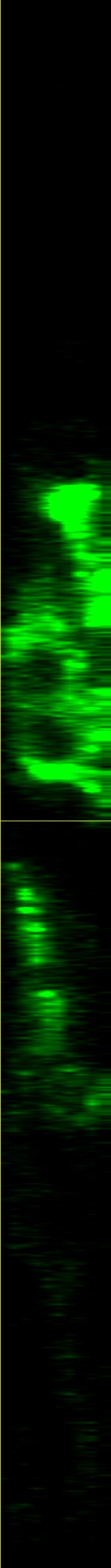

Supplement: Supplementary file 7 — Source Data for Figure 3 [file EMMM-13-e13259-s005.zip › Figure 3/YZ 1924-1 32khz.jpg]

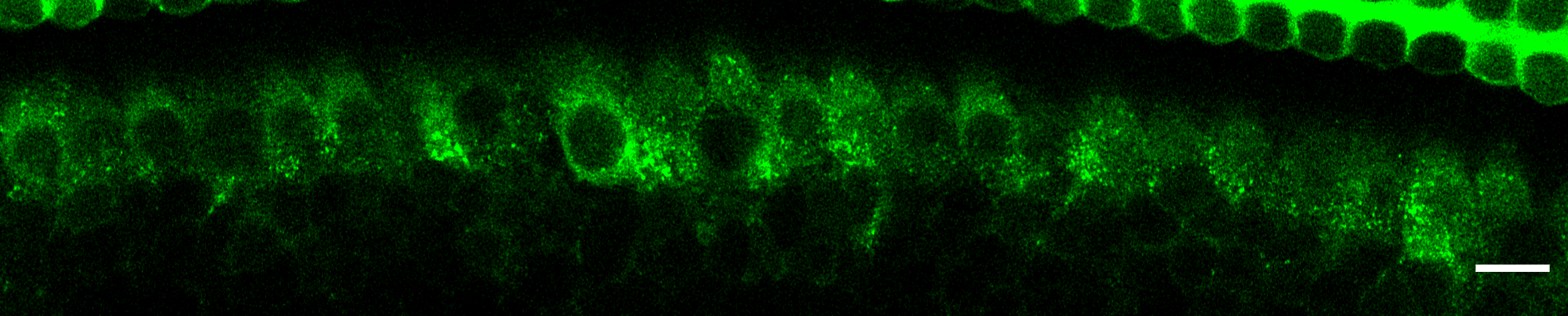

Supplement: Supplementary file 7 — Source Data for Figure 3 [file EMMM-13-e13259-s005.zip › Figure 3/GFP #1 b.27.05 ext 03.06 8khz-2 IHC.png]

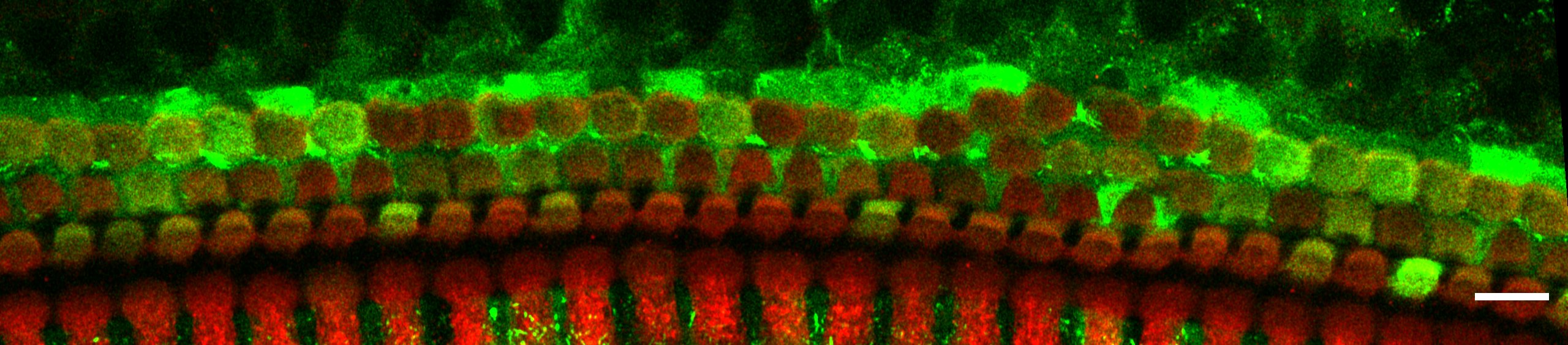

Supplement: Supplementary file 7 — Source Data for Figure 3 [file EMMM-13-e13259-s005.zip › Figure 3/GFP #3 b.27.05 ext 03.06 32khz-1 OHC.czi (RGB).jpg]

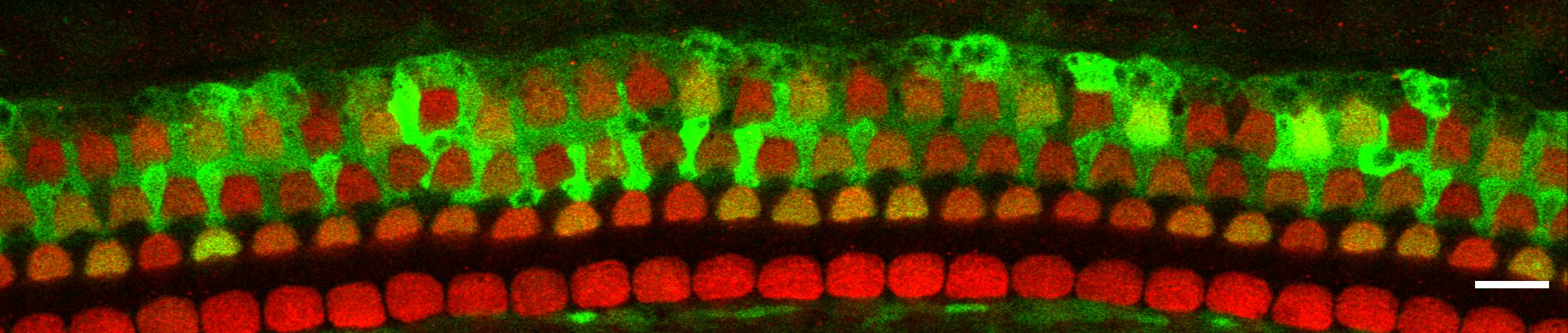

Supplement: Supplementary file 7 — Source Data for Figure 3 [file EMMM-13-e13259-s005.zip › Figure 3/GFP #3 b.27.05 ext 03.06 16khz-1 OHC.czi (RGB).jpg]

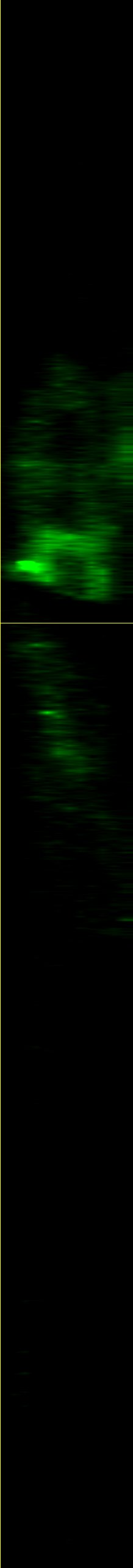

Supplement: Supplementary file 7 — Source Data for Figure 3 [file EMMM-13-e13259-s005.zip › Figure 3/YZ 927-3 8khz.jpg]

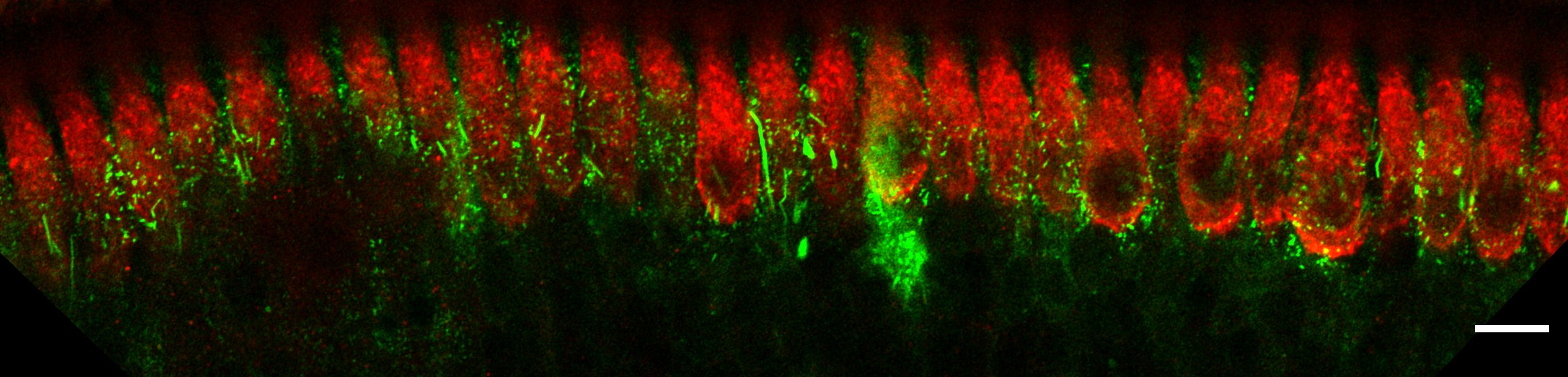

Supplement: Supplementary file 7 — Source Data for Figure 3 [file EMMM-13-e13259-s005.zip › Figure 3/GFP #2 b.27.05 ext 03.06 32khz-1 IHC.czi (RGB).jpg]

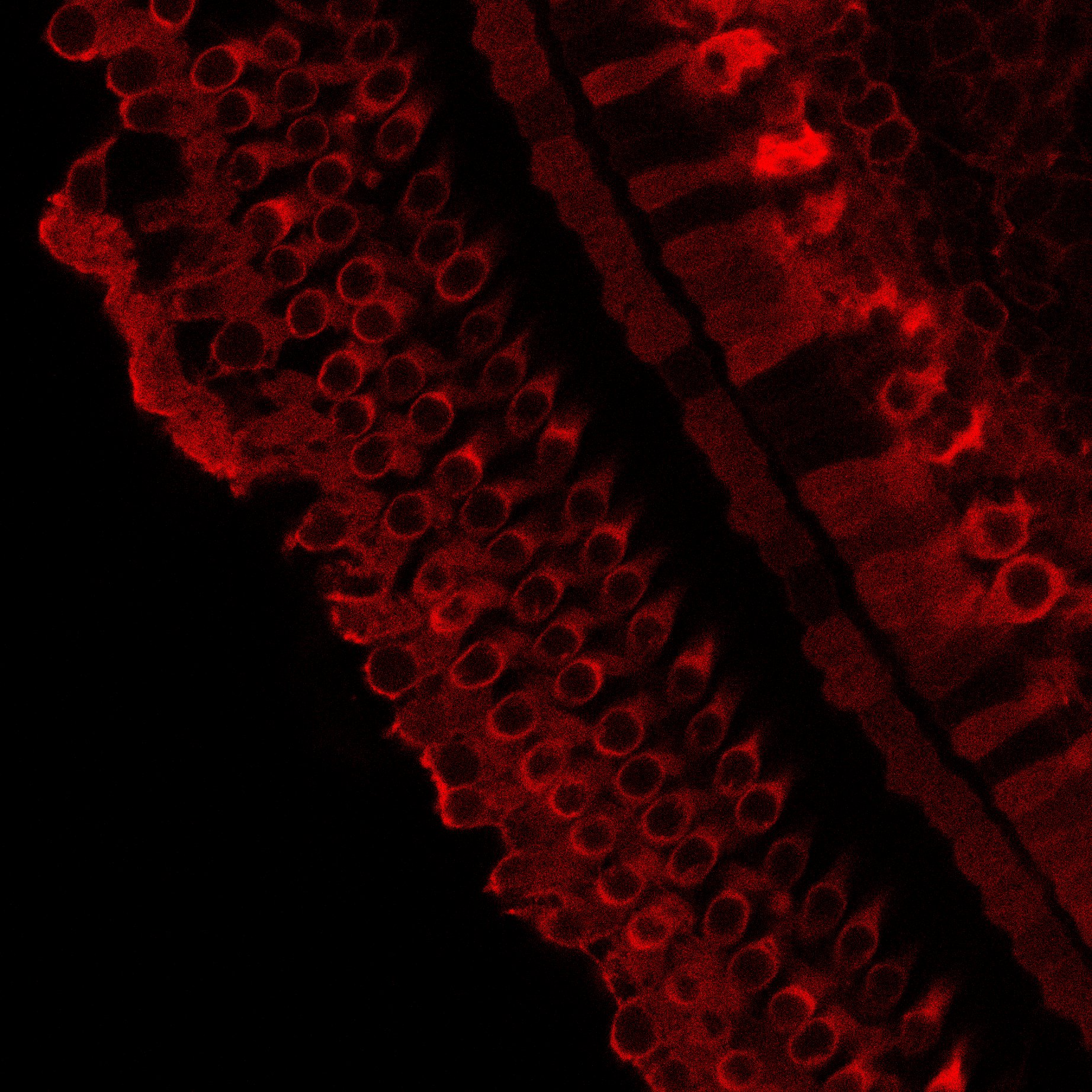

Supplement: Supplementary file 7 — Source Data for Figure 3 [file EMMM-13-e13259-s005.zip › Figure 3/P14 16khz mut inj AAV-S4 FLAG.jpg]

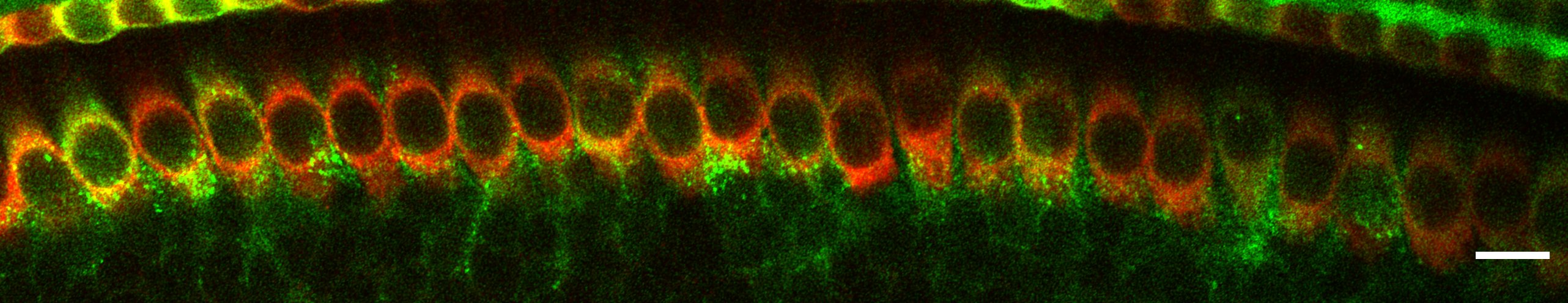

Supplement: Supplementary file 7 — Source Data for Figure 3 [file EMMM-13-e13259-s005.zip › Figure 3/GFP #3 b.27.05 ext 03.06 8khz-1 IHC.czi (RGB).jpg]

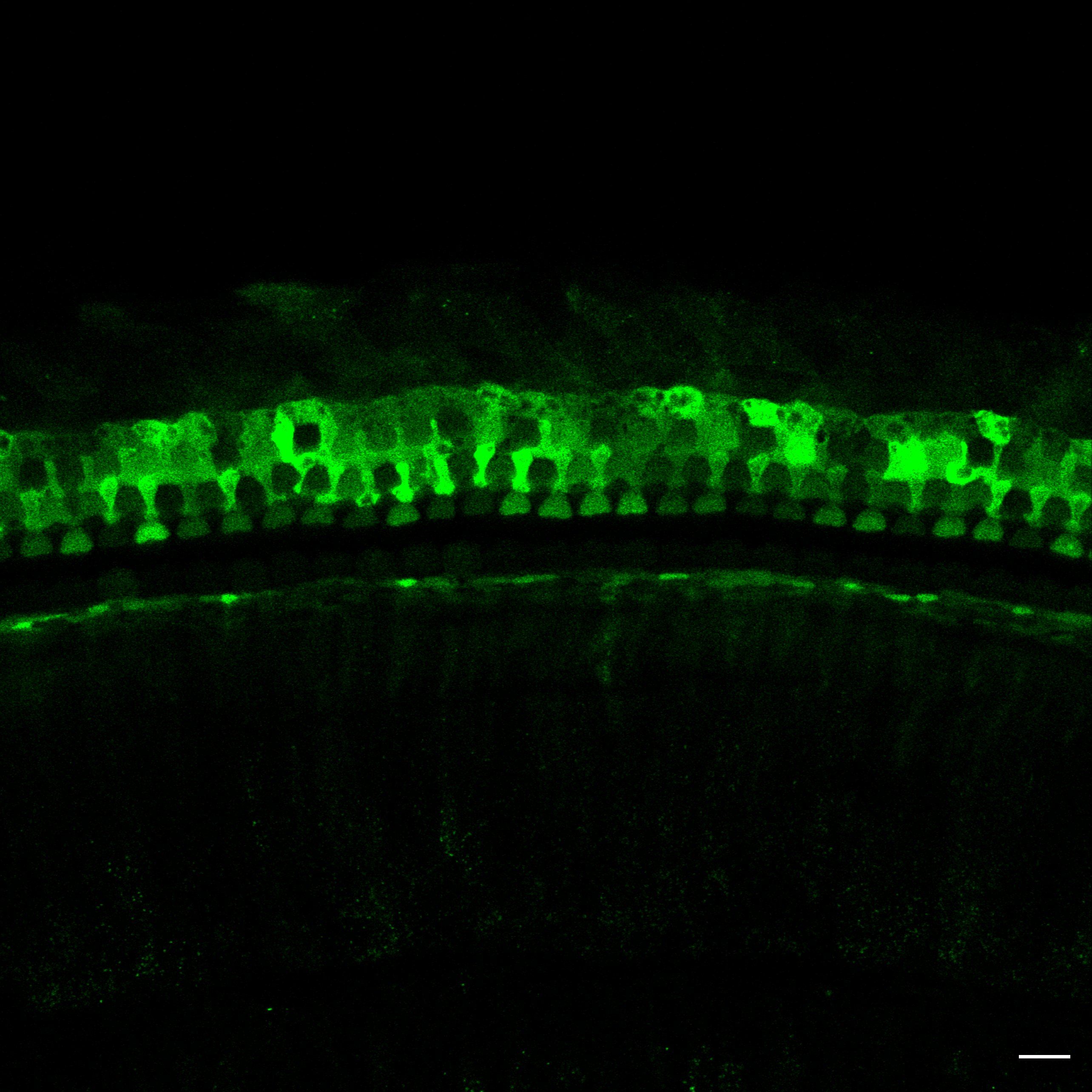

Supplement: Supplementary file 7 — Source Data for Figure 3 [file EMMM-13-e13259-s005.zip › Figure 3/P9 inj AAV-GFP 16khz.jpg]

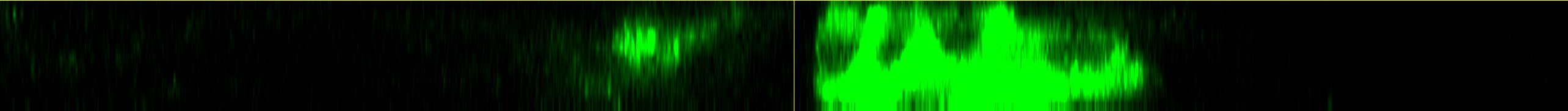

Supplement: Supplementary file 7 — Source Data for Figure 3 [file EMMM-13-e13259-s005.zip › Figure 3/XZ 1200-1 16khz.jpg]

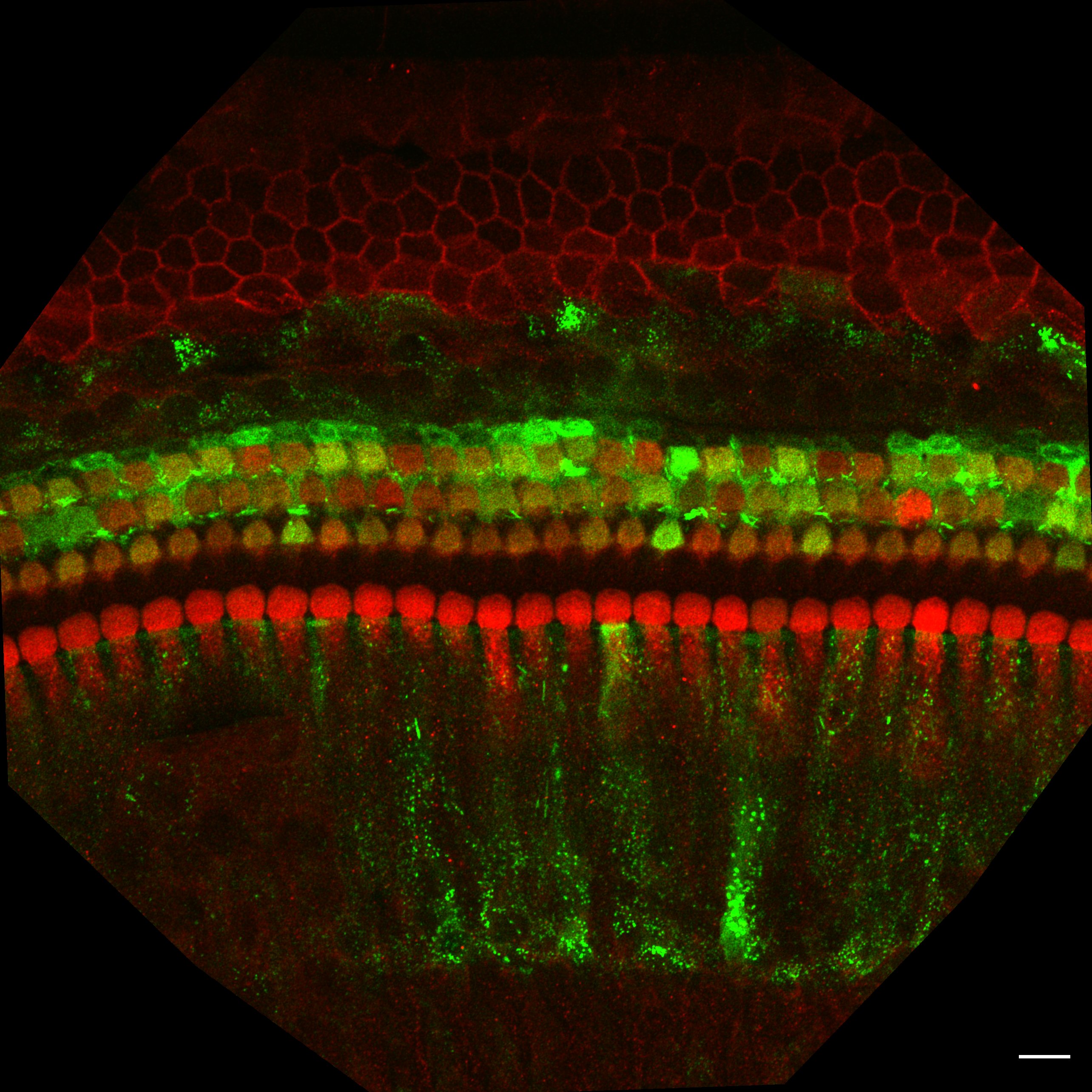

Supplement: Supplementary file 7 — Source Data for Figure 3 [file EMMM-13-e13259-s005.zip › Figure 3/P9 inj AAV-GFP 32khz.jpg]

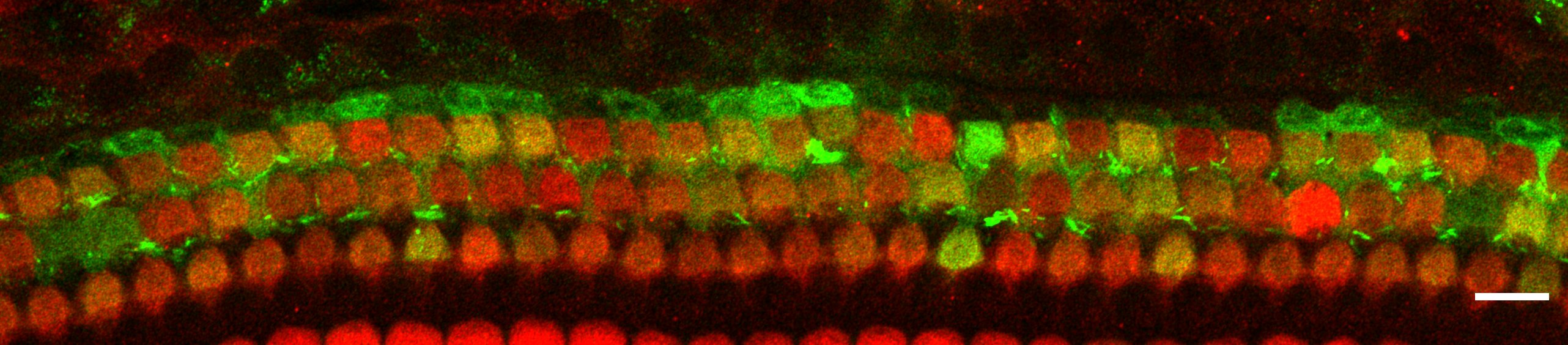

Supplement: Supplementary file 7 — Source Data for Figure 3 [file EMMM-13-e13259-s005.zip › Figure 3/GFP #2 b.27.05 ext 03.06 32khz-1 OHC.czi (RGB).jpg]

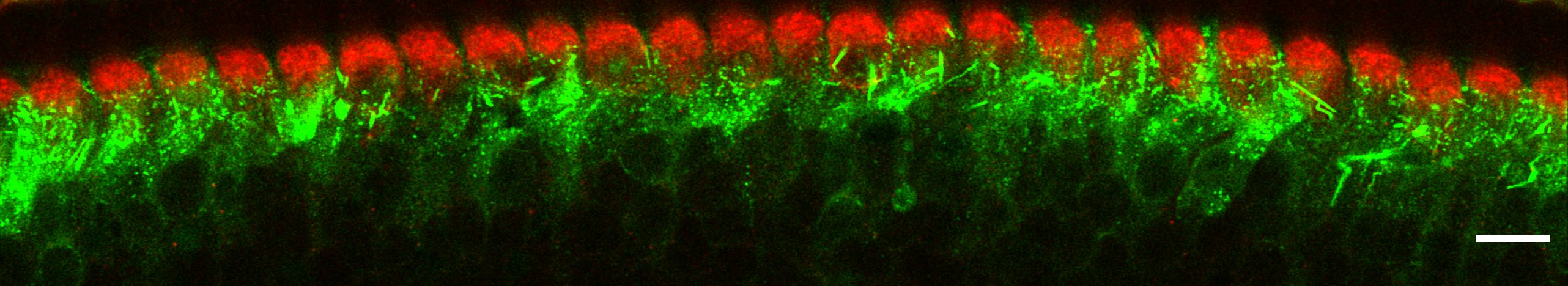

Supplement: Supplementary file 7 — Source Data for Figure 3 [file EMMM-13-e13259-s005.zip › Figure 3/GFP #3 b.27.05 ext 03.06 16khz-1 IHC.czi (RGB).jpg]

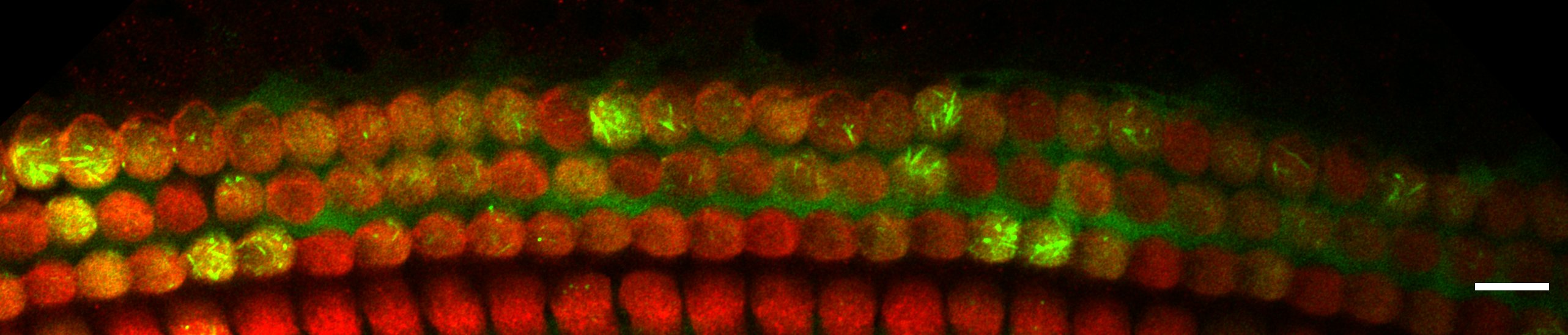

Supplement: Supplementary file 7 — Source Data for Figure 3 [file EMMM-13-e13259-s005.zip › Figure 3/GFP #3 b.27.05 ext 03.06 8khz-1 OHC.czi (RGB).jpg]

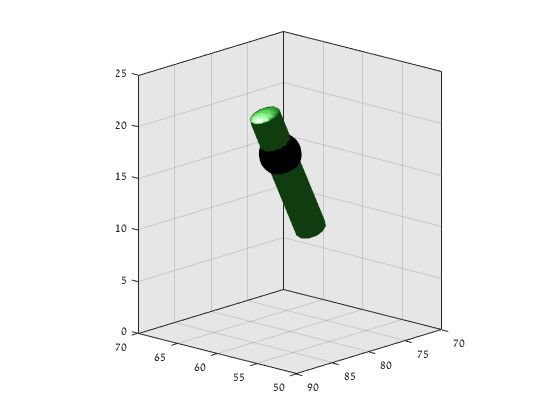

Supplement: Supplementary file 8 — Source Data for Figure 4 [file EMMM-13-e13259-s006.zip › Figure 4/P14 8khz mut OHC example.png]

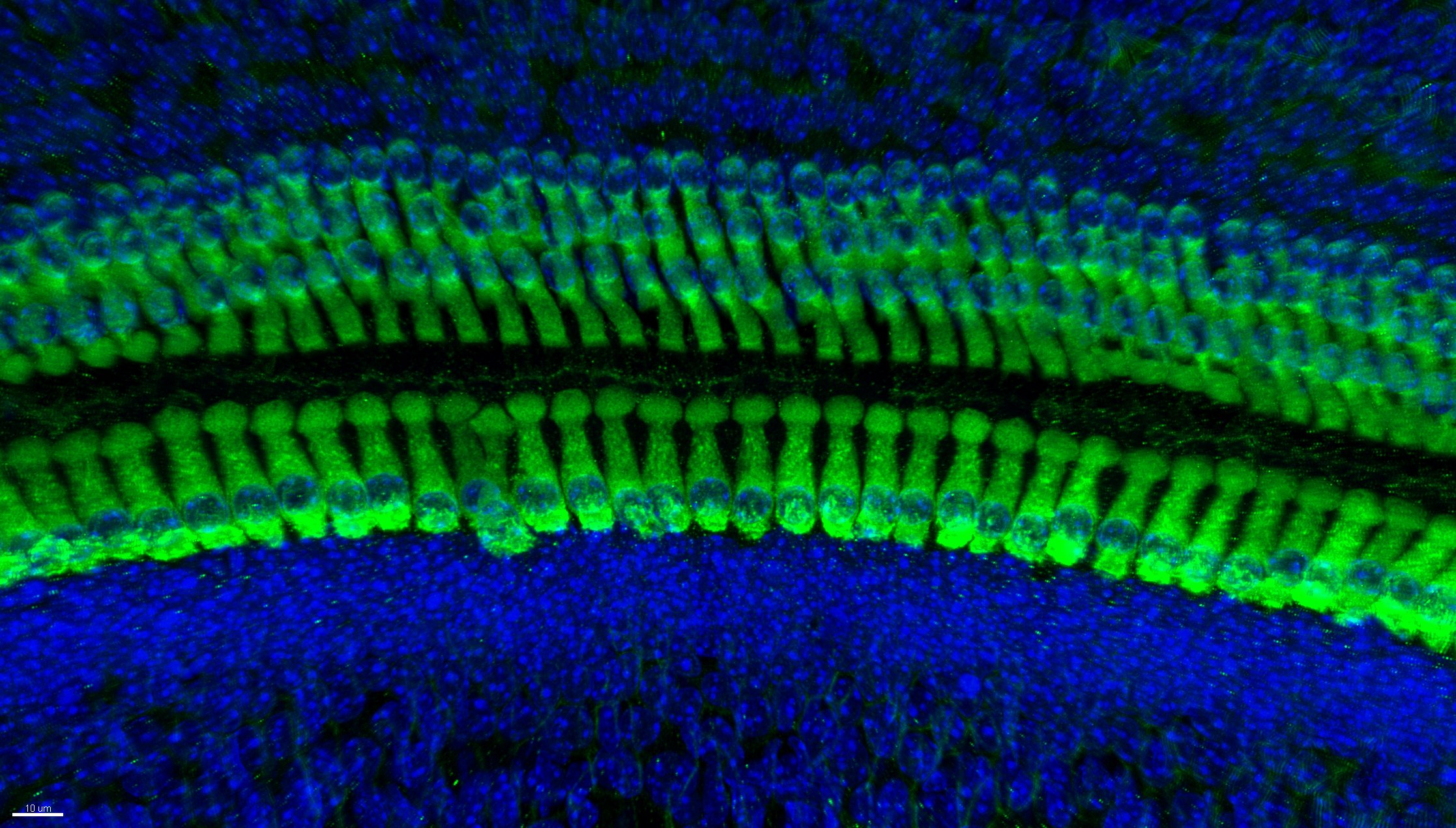

Supplement: Supplementary file 8 — Source Data for Figure 4 [file EMMM-13-e13259-s006.zip › Figure 4/P14 8khz WT nuc pos.jpg]

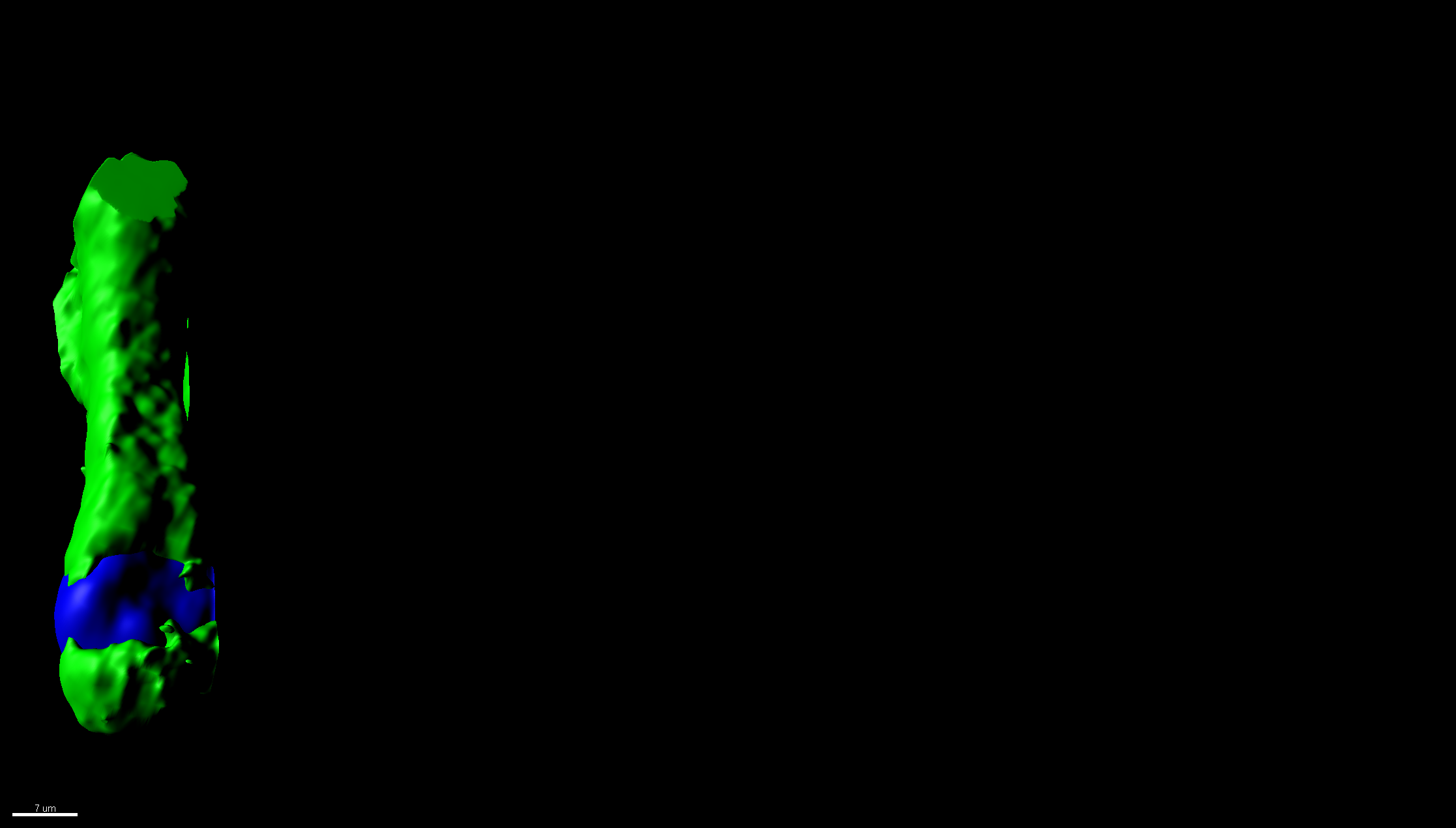

Supplement: Supplementary file 8 — Source Data for Figure 4 [file EMMM-13-e13259-s006.zip › Figure 4/P14 8khz mut inj AAV-S4 nuc pos OHC surface.tif]

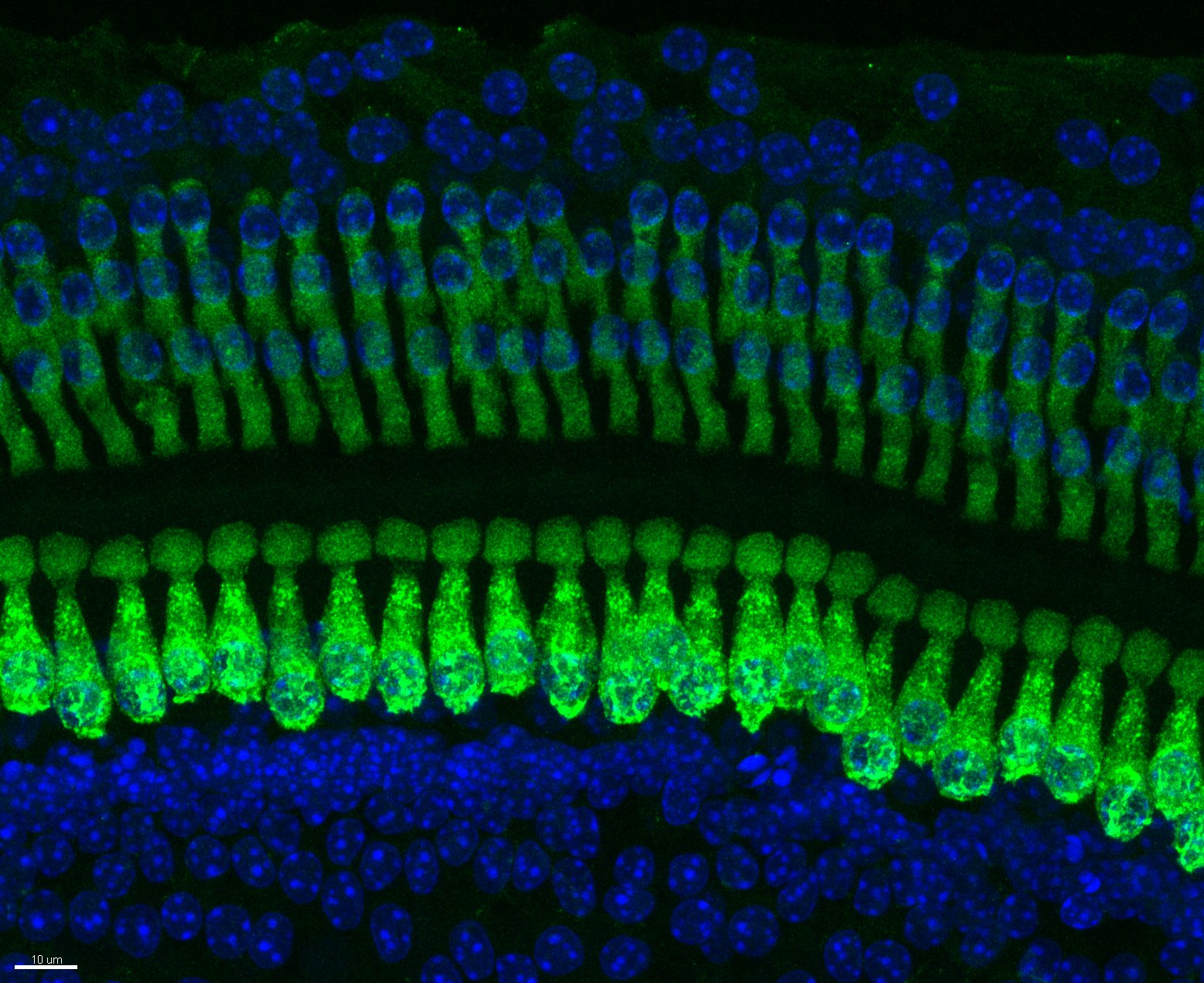

Supplement: Supplementary file 8 — Source Data for Figure 4 [file EMMM-13-e13259-s006.zip › Figure 4/P14 8khz mut inj AAV-S4 nuc pos.jpg]

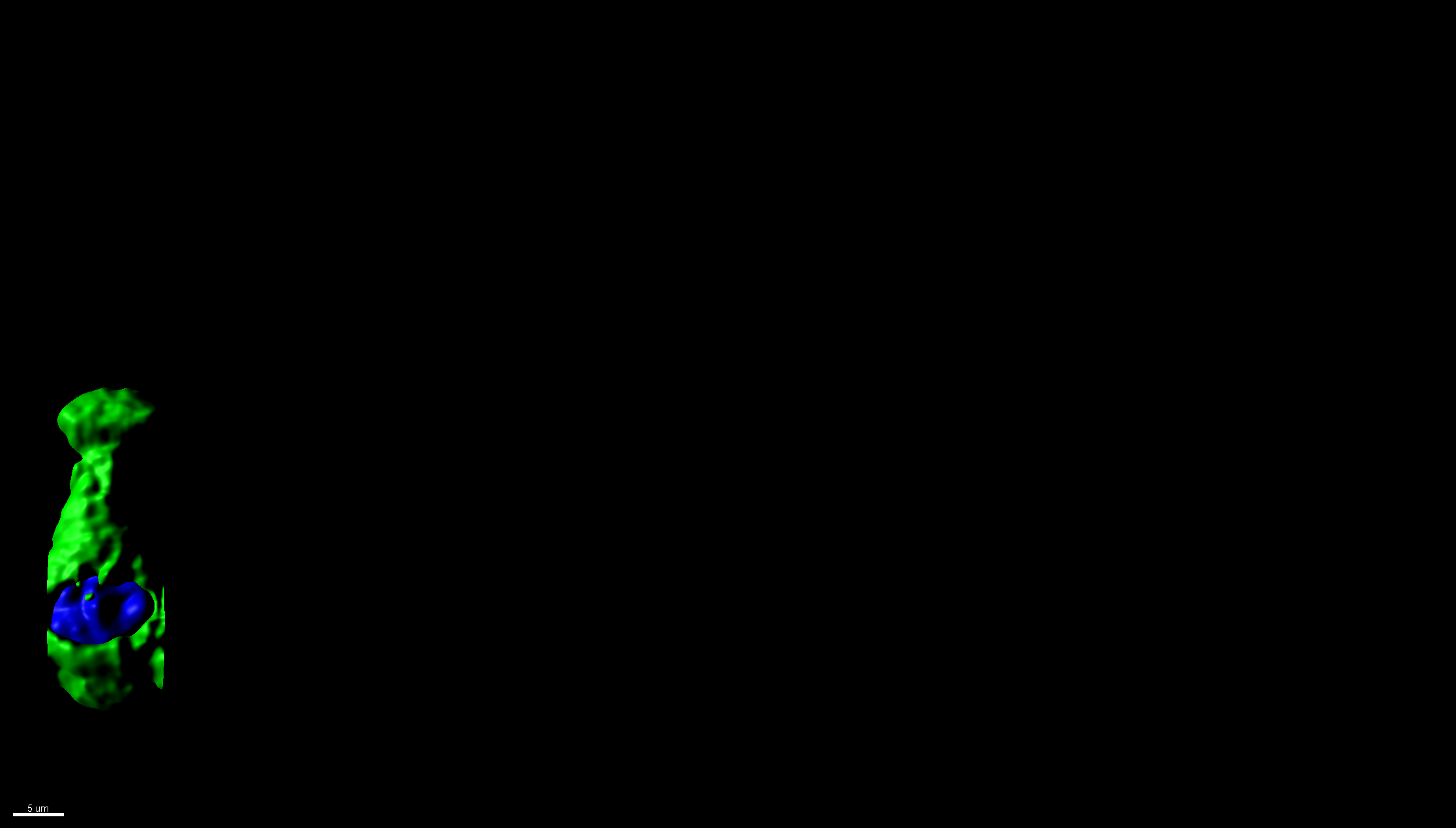

Supplement: Supplementary file 8 — Source Data for Figure 4 [file EMMM-13-e13259-s006.zip › Figure 4/P14 8khz WT nuc pos IHC surface.tif]

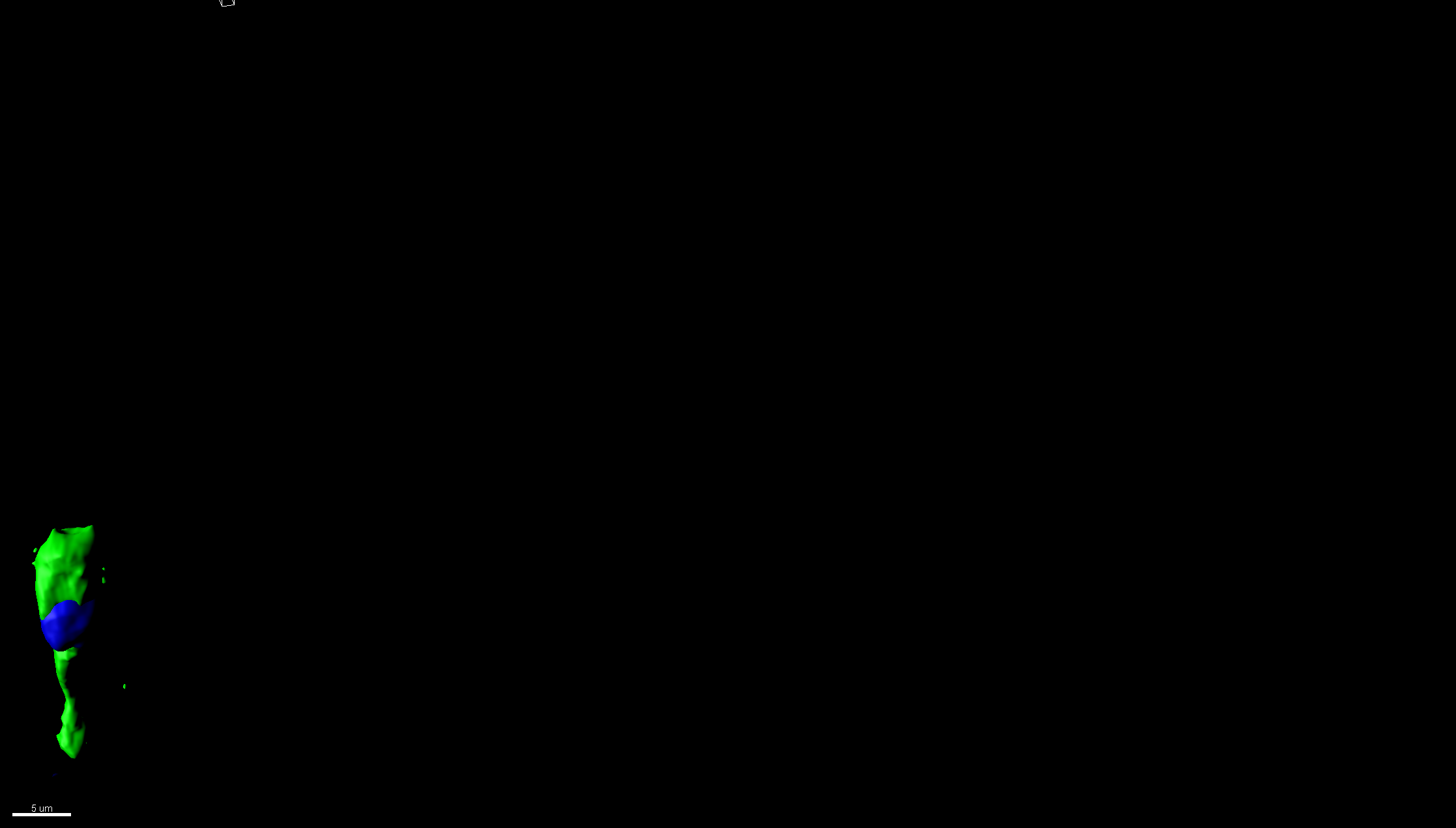

Supplement: Supplementary file 8 — Source Data for Figure 4 [file EMMM-13-e13259-s006.zip › Figure 4/P14 8khz mut nuc pos OHC surface.tif]

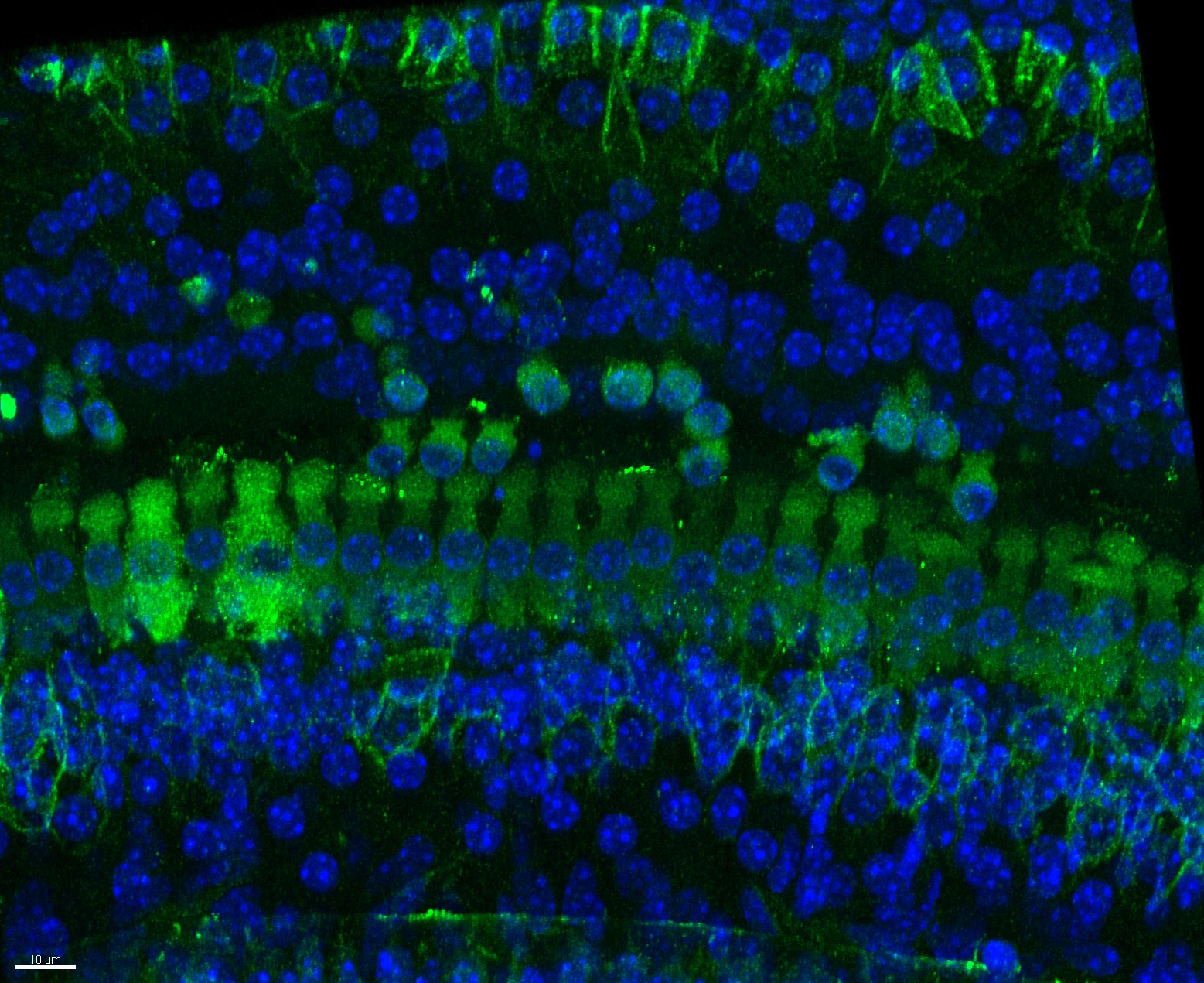

Supplement: Supplementary file 8 — Source Data for Figure 4 [file EMMM-13-e13259-s006.zip › Figure 4/P14 16khz mut nuc pos.jpg]

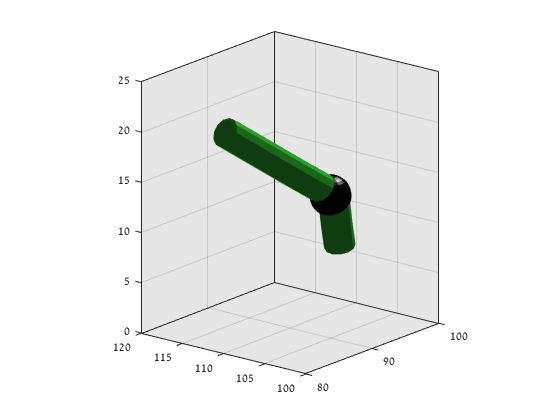

Supplement: Supplementary file 8 — Source Data for Figure 4 [file EMMM-13-e13259-s006.zip › Figure 4/P14 8khz mut inj OHC.png]

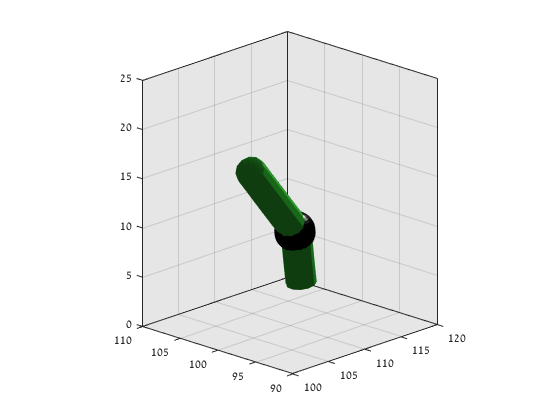

Supplement: Supplementary file 8 — Source Data for Figure 4 [file EMMM-13-e13259-s006.zip › Figure 4/P14 8khz wt OHC example.png]

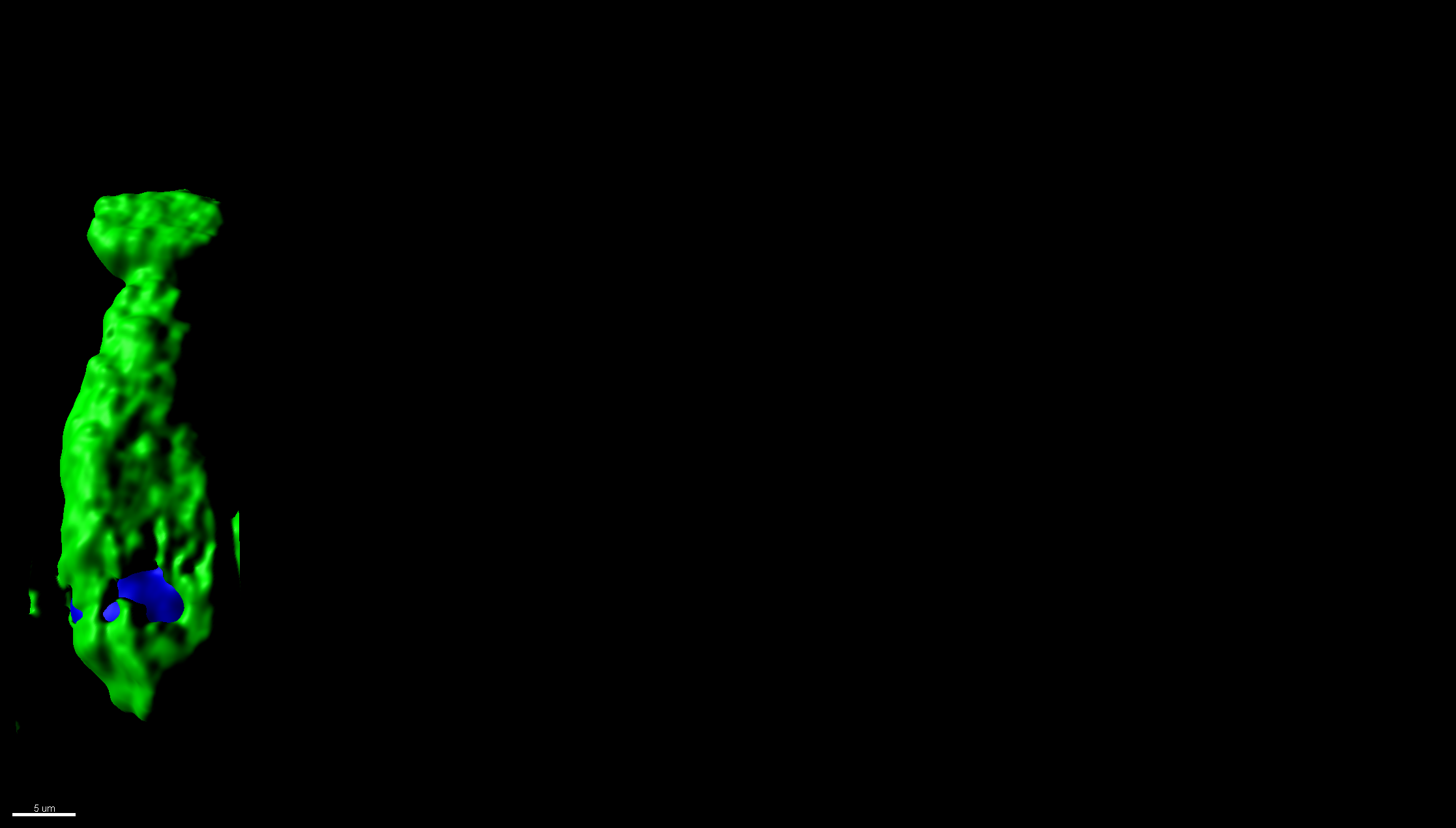

Supplement: Supplementary file 8 — Source Data for Figure 4 [file EMMM-13-e13259-s006.zip › Figure 4/P14 8khz mut inj AAV-S4 nuc pos IHC surface.tif]

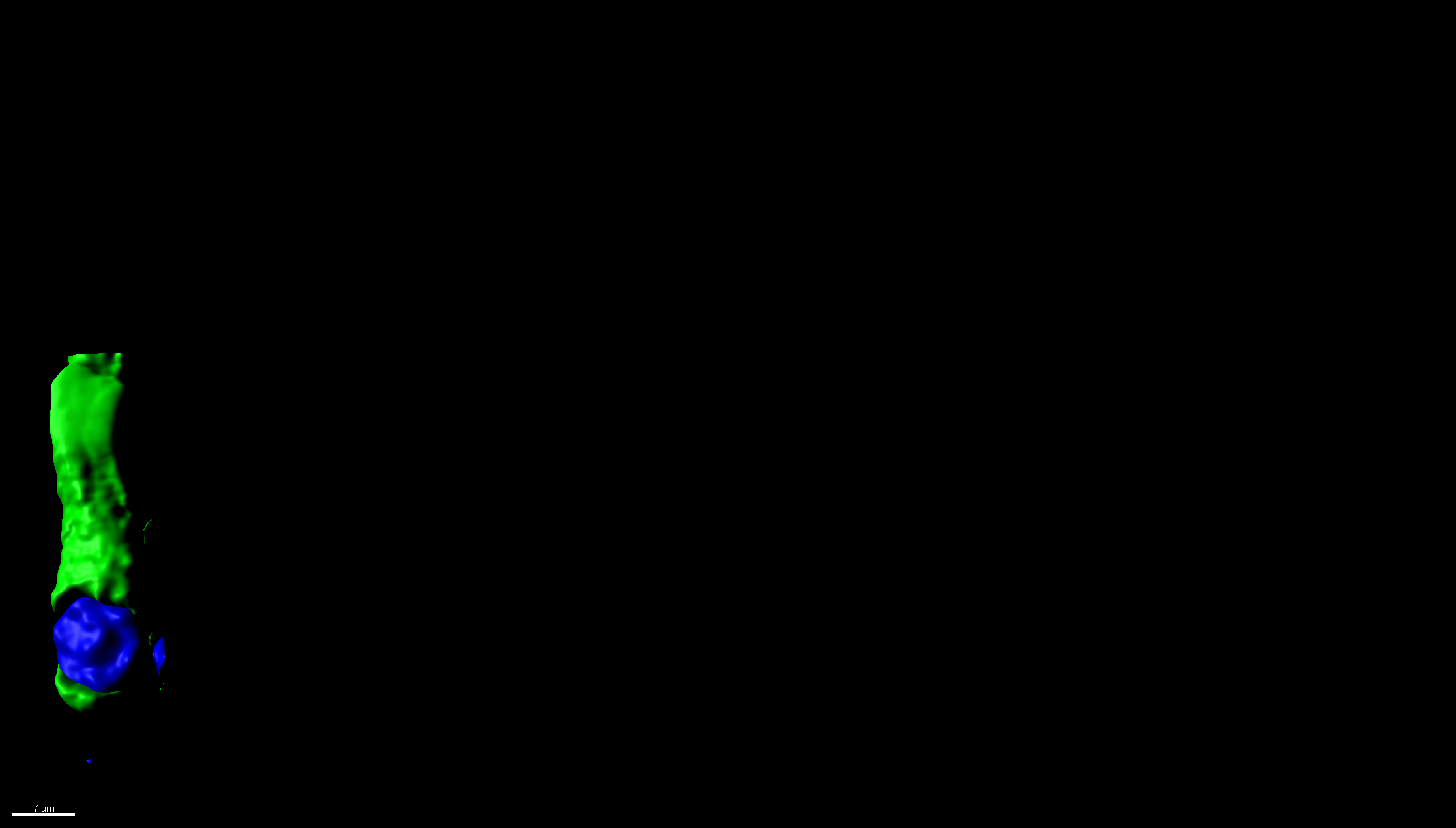

Supplement: Supplementary file 8 — Source Data for Figure 4 [file EMMM-13-e13259-s006.zip › Figure 4/P14 8khz WT nuc pos OHC surface.tif]

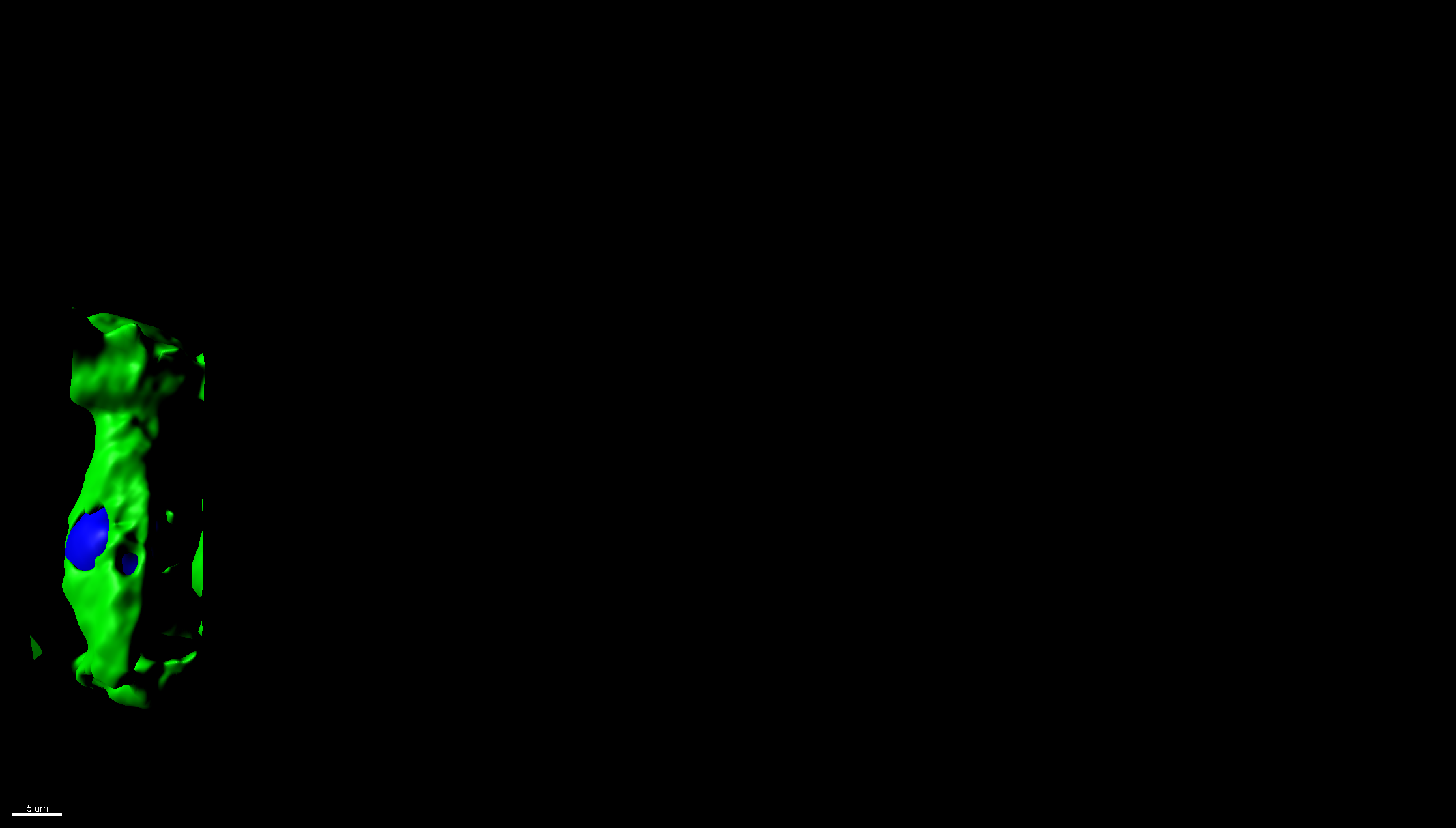

Supplement: Supplementary file 8 — Source Data for Figure 4 [file EMMM-13-e13259-s006.zip › Figure 4/P14 8khz mut nuc pos IHC surface.tif]

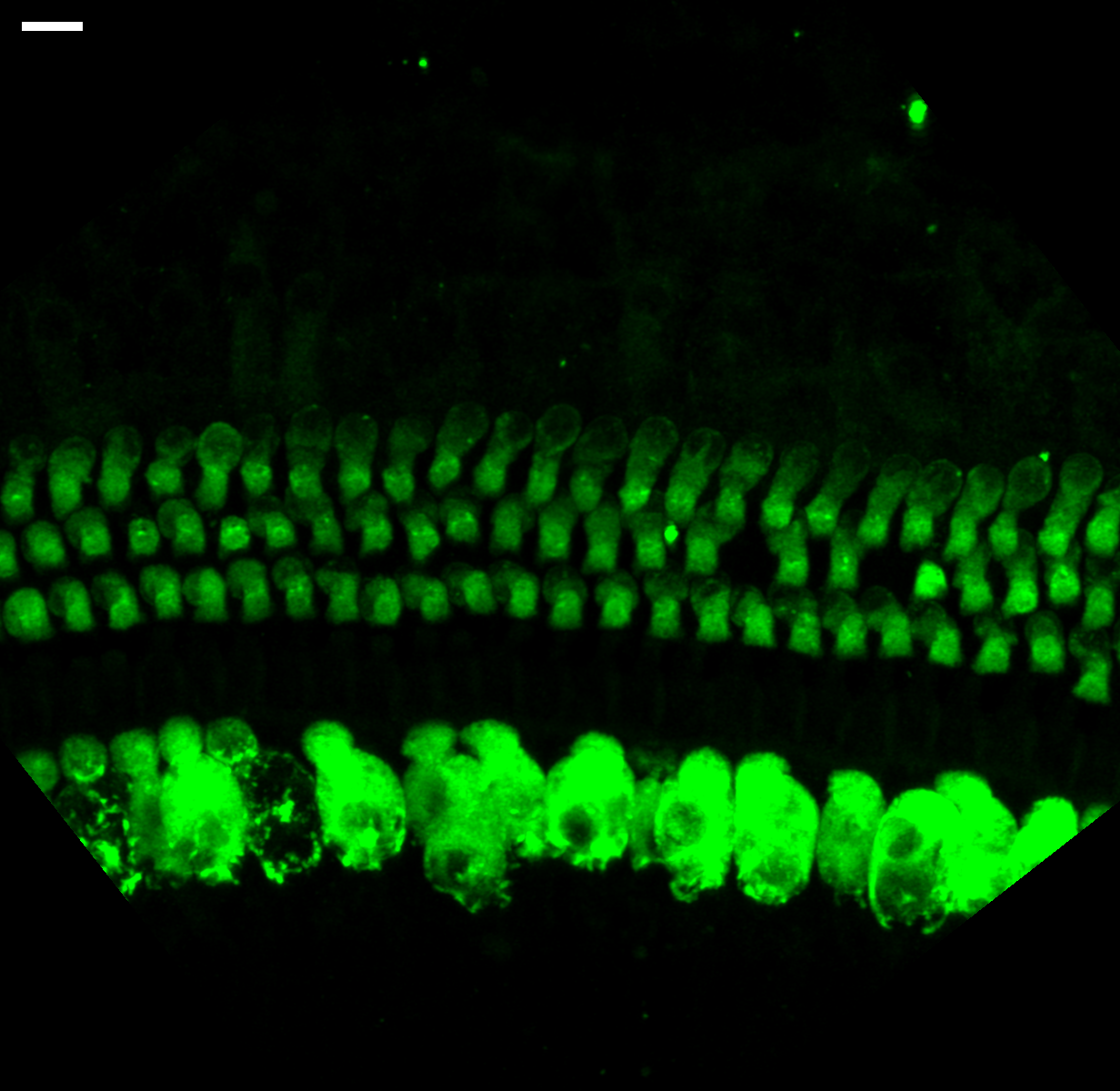

Supplement: Supplementary file 10 — Source Data for Figure 6 [file EMMM-13-e13259-s008.zip › Figure 6/12w mut inj AAV-S4 12khz.png]

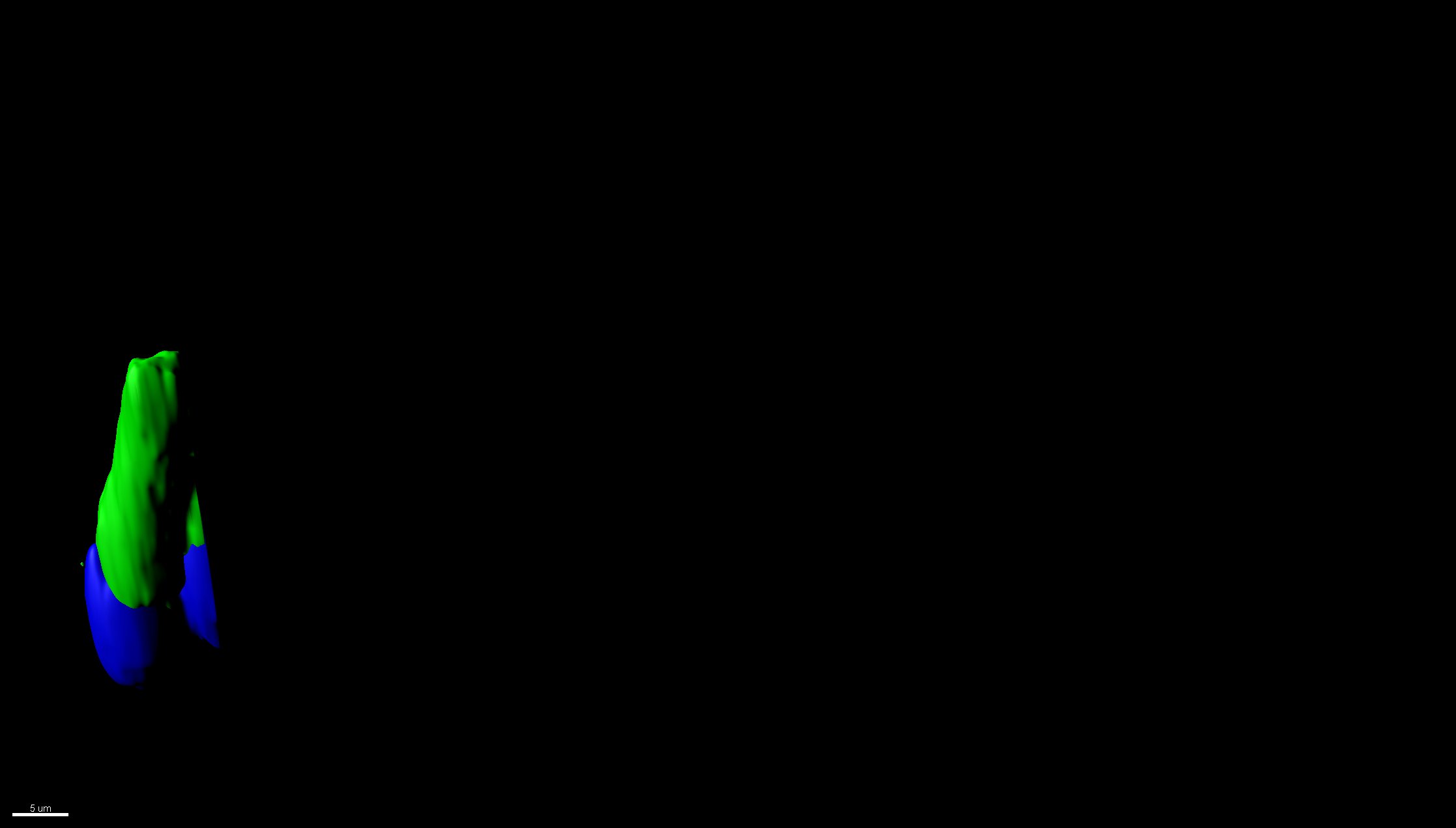

Supplement: Supplementary file 10 — Source Data for Figure 6 [file EMMM-13-e13259-s008.zip › Figure 6/12w WT nuc pos OHC surface.jpg]

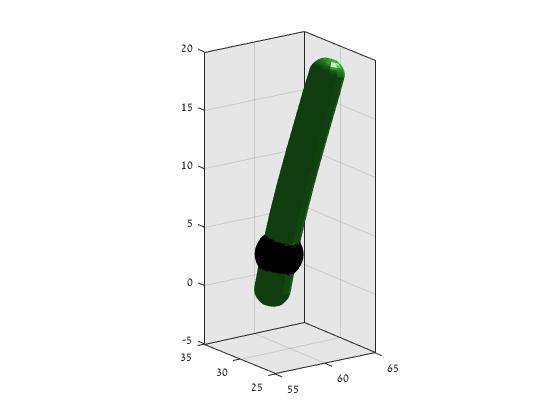

Supplement: Supplementary file 10 — Source Data for Figure 6 [file EMMM-13-e13259-s008.zip › Figure 6/12w mut inj AAV-S4 OHC example.jpg]

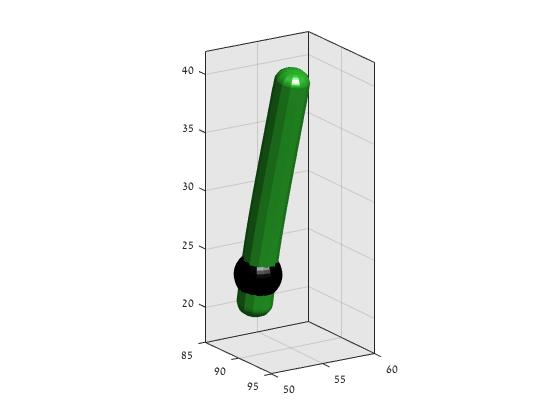

Supplement: Supplementary file 10 — Source Data for Figure 6 [file EMMM-13-e13259-s008.zip › Figure 6/12w WT OHC example.jpg]

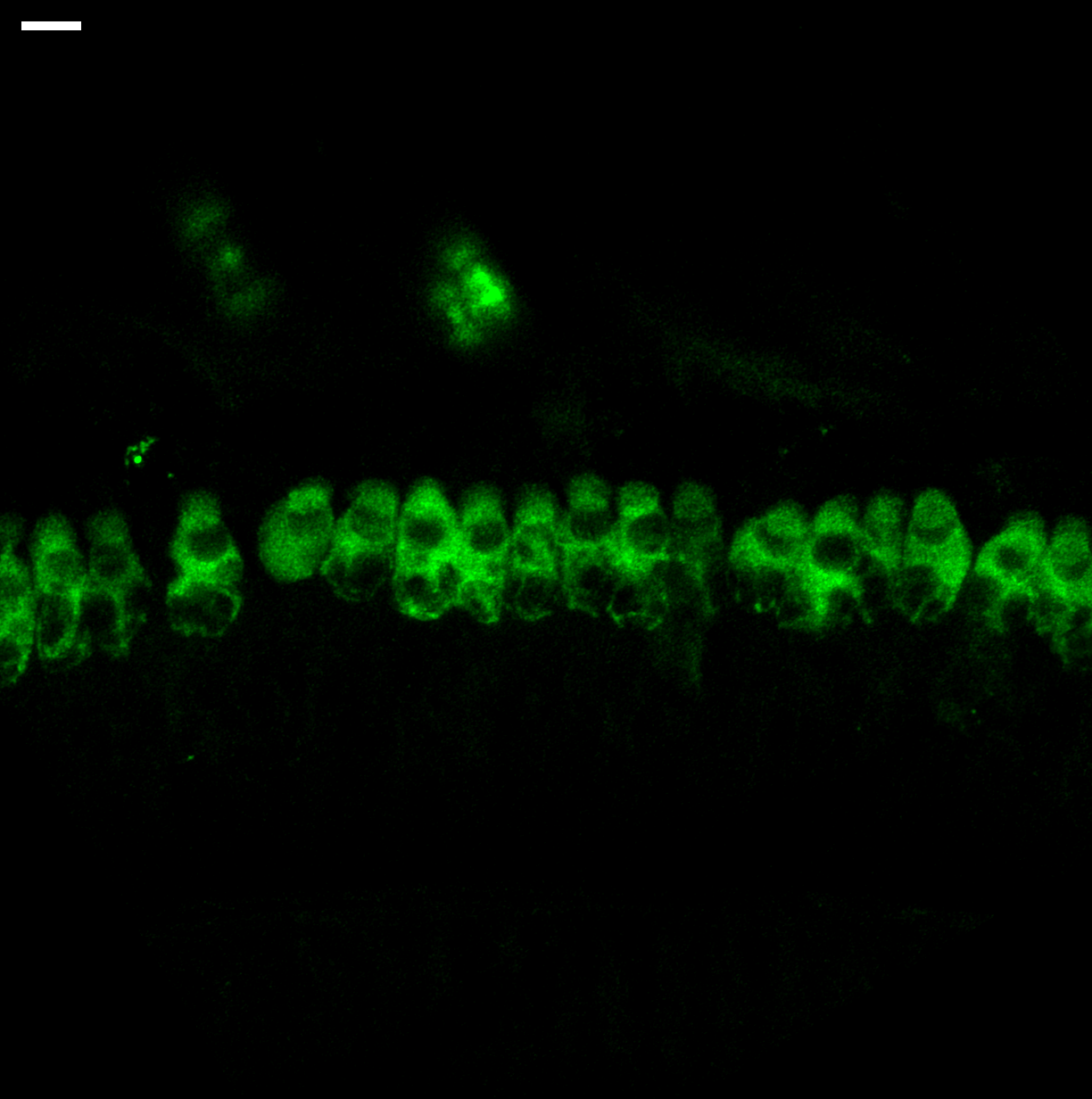

Supplement: Supplementary file 10 — Source Data for Figure 6 [file EMMM-13-e13259-s008.zip › Figure 6/12w mut 12khz.png]

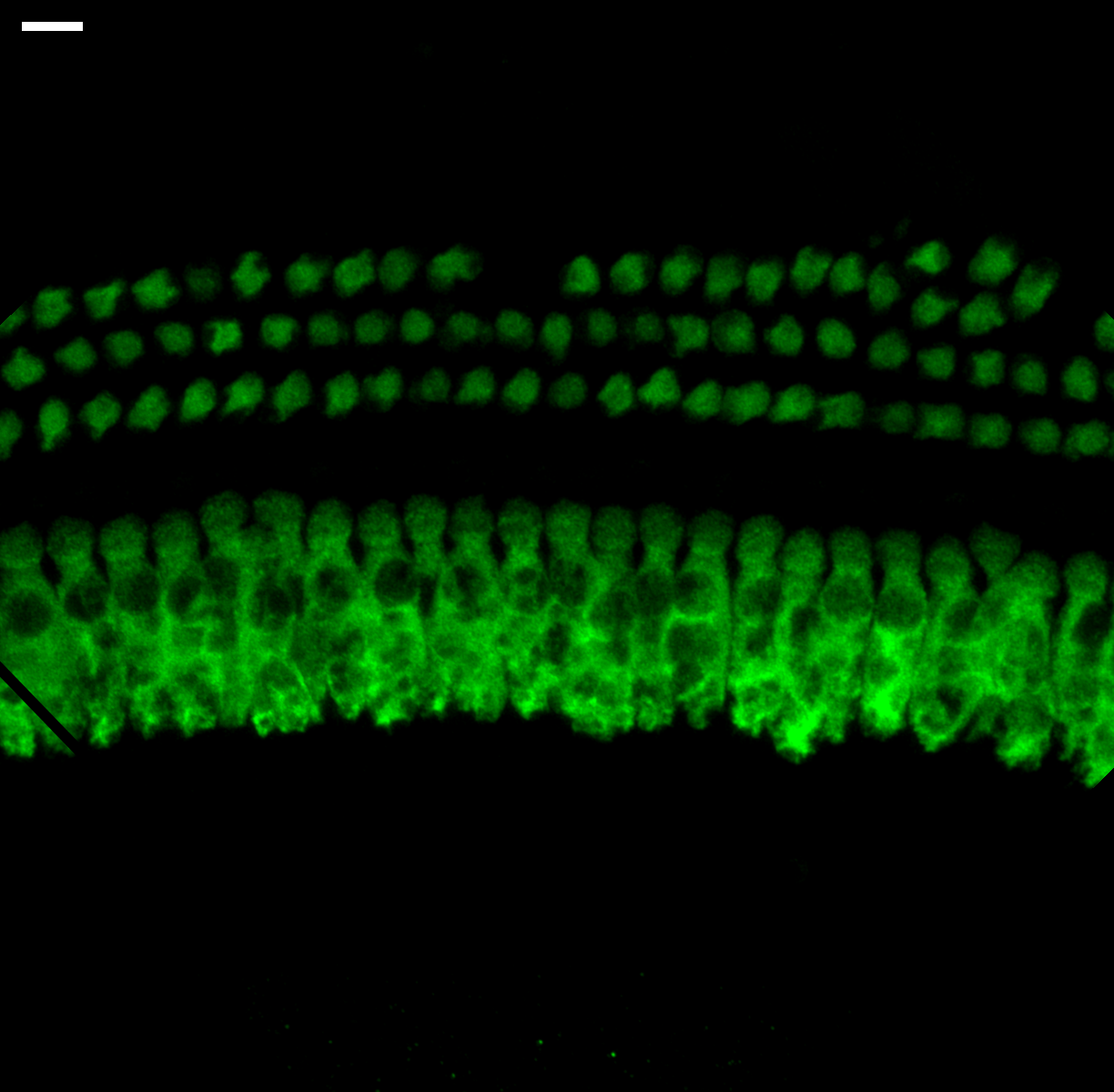

Supplement: Supplementary file 10 — Source Data for Figure 6 [file EMMM-13-e13259-s008.zip › Figure 6/12w WT 12khz.png]

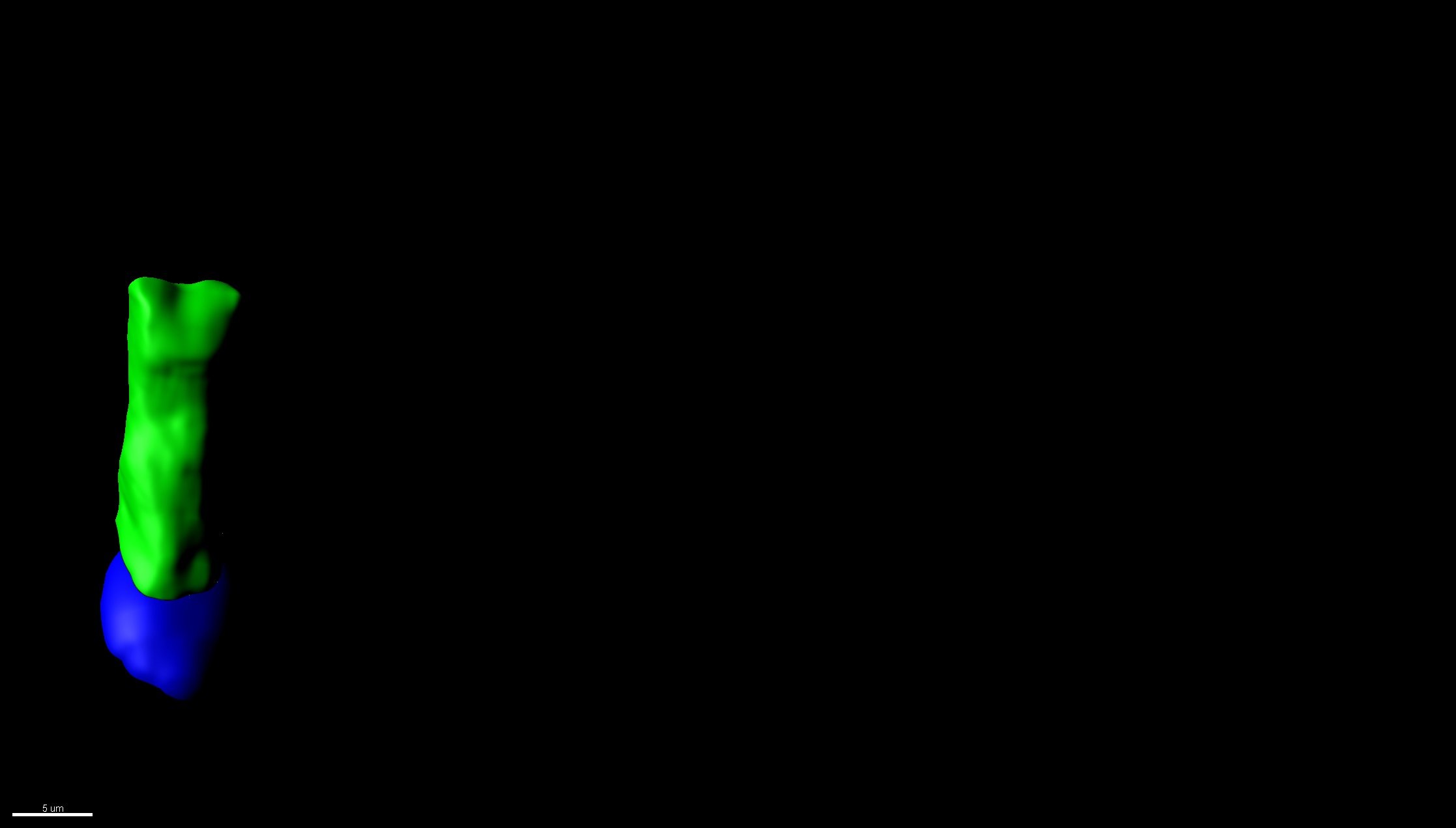

Supplement: Supplementary file 10 — Source Data for Figure 6 [file EMMM-13-e13259-s008.zip › Figure 6/12w mut inj AAV-S4 nuc pos OHC surface.jpg]
